# Visualizing a Li‐Depleted Amorphous Cathode–Electrolyte Interphase in Sulfide Solid‐State Batteries Using In Situ Cryogenic Electron Microscopy

**DOI:** 10.1002/advs.77717

**Published:** 2026-09-13

**Authors:** Yuki Nomura, Ryoma Sasaki, Huu Duc Luong, Misaki Hasegawa, Willy Shun Kai Bong, Koji Hiraoka, Kazuo Yamamoto, Yutaka Ito, Yoshiya Fujiwara, Yoshitaka Tateyama, Takuhiro Miyuki

**Affiliations:** ^1^ Nanostructures Research Laboratory Japan Fine Ceramics Center Nagoya Aichi Japan; ^2^ Laboratory for Chemistry and Life Science Institute of Integrated Research Institute of Science Tokyo Yokohama Kanagawa Japan; ^3^ Consortium for Lithium Ion Battery Technology and Evaluation Research Center Ikeda Osaka Japan

**Keywords:** electrochemical degradation, electron energy‐loss spectroscopy, interfacial resistance, low dose, machine‐learning potential molecular dynamics simulations, scanning nanobeam electron diffraction, scanning transmission electron microscopy

## Abstract

Electrochemical degradation at the cathode/solid‐electrolyte interface critically limits the performance of sulfide‐based solid‐state batteries; however, its nanoscale origin remains unclear. Here, we directly visualize the evolution of Li distribution and crystal structure at the LiNi_0.5_Co_0.2_Mn_0.3_O_2_/Li_6_PS_5_Cl interface during charging using in situ scanning transmission electron microscopy, combined with electron energy‐loss spectroscopy and energy‐filtered nanobeam electron diffraction. A potential‐controlled sample‐preparation protocol is developed to preserve the native interphase structure during sample preparation, and low‐dose‐rate cryogenic electron microscopy minimizes electron‐beam‐induced damage. At the uncoated interface, charging induced the formation of a ∼50‐nm‐thick Li‐depleted interphase. Within ∼20 nm of the interface, the Li concentration decreased to below *x* = 4 in Li*_x_* PS_5_Cl, accompanied by amorphization of the solid electrolyte. Machine‐learning‐potential molecular dynamics simulations reveal that Li depletion destabilizes the argyrodite Li_6_PS_5_Cl framework and increases the Li‐ion migration barrier, providing microscopic insight into the increase in interfacial resistance. Conversely, LiNbO*_y_*‐coated interfaces exhibited neither pronounced Li depletion nor significant amorphization, even with coating thicknesses as small as 5 nm, thereby elucidating the buffering mechanism of LiNbO*_y_* at the cathode/sulfide‐solid‐electrolyte interface. These results establish Li‐depletion‐induced structural disorder as an important transport‐limiting mechanism at cathode interfaces employing sulfide solid electrolytes, providing a framework for interface engineering.

## Graphical abstract

In situ cryogenic STEM‐EELS and electron diffraction directly visualize Li depletion and structural degradation at the NCM523/Li_6_PS_5_Cl interface during charging. The results reveal a Li‐depleted amorphous interphase that impedes Li transport, while LiNbO*_y_* coating suppresses interfacial degradation and preserves the solid‐electrolyte structure.

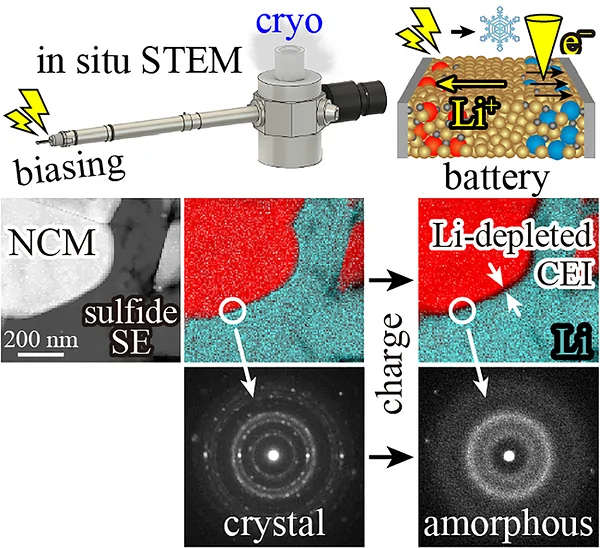

## 1 Introduction

Solid‐state Li batteries (SSBs) have emerged as a promising platform for the advancement of battery technology [1, 2]. Sulfide solid electrolytes (SEs) have attracted considerable attention because of their high ionic conductivity and good deformability [3, 4, 5, 6, 7, 8, 9]. However, their narrow electrochemical stability window (typically 1.5–2.5 V vs. Li) leads to oxidative and reductive decomposition at interfaces [10, 11, 12, 13, 14, 15]. This decomposition results in the loss of physical contact and the formation of decomposition‐product layers, referred to as the cathode–electrolyte interphase (CEI) on the cathode side; both effects contribute to an increase in interfacial resistance. To mitigate this oxidative decomposition, electronically insulating coating layers such as LiNbO*_y_* are widely used [16]. However, interfacial resistance still increases under high‐voltage charging [17], elevated temperatures [18], extended cycling [12], or with specific cathode materials such as LiFePO_4_ [19]. Although thicker coatings can effectively suppress decomposition, the resulting excessive thickness impedes electron transport within the cathode composite and ion transport across the interface. Moreover, cathode coating layers often exhibit pronounced nanoscale thickness variations. Consequently, the extent to which reduced interfacial resistance arises from improved coverage versus increased local thickness remains unclear. Furthermore, reports on the critical coating thickness required to effectively suppress decomposition remain limited [20, 21].

A central unresolved issue in understanding CEI formation is the absence of direct microscopic insight into its early‐stage formation and growth. In particular, the spatial distribution of Li, which directly governs ionic transport and interfacial resistance, has not been resolved experimentally at the nanoscale. Two primary mechanisms have been proposed to explain interfacial resistance growth in sulfide‐based SSBs: intrinsic oxidative decomposition of the sulfide SE and chemically driven reactions between the sulfide SE and oxygen that is released from the highly charged cathode, which typically occurs above ∼4 V vs. Li. X‐ray photoelectron spectroscopy (XPS) studies have demonstrated that sulfide SEs undergo intrinsic oxidative decomposition at potentials above ∼2.8 V vs. Li [22], which is well below the operating voltages of typical oxide cathodes. Electrochemical impedance spectroscopy (EIS) measurements also show that the interfacial resistance begins to increase even below 4 V vs. Li [12]. These results suggest that intrinsic SE decomposition plays a dominant role in the early stages of CEI formation and the accompanying increase in interfacial resistance. However, because XPS probes spatially averaged information, it can neither determine the interfacial location of such decomposition nor resolve the thickness and morphology of the CEI. In contrast, time‐of‐flight secondary‐ion mass spectrometry (ToF‐SIMS) and scanning transmission X‐ray microscopy (STXM) provide spatially resolved chemical mapping across the cathode/SE interface but predominantly detect µm‐scale PO*_x_* and SO*_x_* species that are associated with the reactions involving highly charged cathodes or prolonged cycling [23, 24, 25]. Accordingly, ToF‐SIMS and STXM primarily capture interfacial reactions that develop at later stages, rather than the intrinsic SE decomposition responsible for initiating CEI formation. These techniques are also limited in their ability to quantitatively resolve Li distributions with sufficient spatial resolution. Consequently, the thickness and morphology of the CEI formed by intrinsic SE decomposition cannot be directly determined, leaving their impact on interfacial resistance growth unclear.

To bridge this gap, microscopy techniques that are capable of directly visualizing the nanoscale structure of the CEI while identifying Li distribution and intrinsic decomposition products of the sulfide SE are required. Such an approach is essential for determining whether intrinsic SE decomposition forms a continuous resistive layer or localized domains, and for elucidating the effect of its composition, crystal structure, and spatial heterogeneity on ion and electron transport across the cathode/SE interface. Scanning transmission electron microscopy (STEM), combined with electron energy‐loss spectroscopy (EELS) and electron diffraction [26], provides the spatial resolution and chemical sensitivity required to identify local CEI structures and resolve the Li distribution, thereby addressing these challenges [27, 28]. However, sulfide SEs are highly susceptible to electron‐beam damage, and even cryogenic conditions cannot completely suppress irradiation‐induced degradation [29, 30, 31]. Moreover, the use of multiple sample preparation steps for STEM, such as focused ion‐beam (FIB) thinning, can alter the local electrostatic potential at the cathode/SE interface and thereby change the CEI structure. Therefore, maintaining the potential distribution and preserving the native CEI structure are critical factors to be considered during both preparation and observation.

Here, we present a potential‑controlled sample preparation technique that preserves the native CEI structure during sample preparation processes, such as FIB thinning. To minimize electron‐beam‐induced damage during interfacial imaging, a combination of low‐dose‐rate and cryogenic STEM was used [32, 33], which substantially reduced irradiation‐induced Li depletion and amorphization during observation. Furthermore, by leveraging an in situ observation technique to compare the same field of view before and after charging [34, 35, 36, 37, 38], clearer microscopic insights into the CEI at the LiNi_0.5_Co_0.2_Mn_0.3_O_2_ (NCM523)/Li_6_PS_5_Cl (LPSC) interface were obtained using STEM‐EELS and energy‐filtered scanning nanobeam electron diffraction (EF‐SNBD). Complementary machine‐learning‐potential molecular dynamics simulations quantitatively evaluated the Li‐ion self‐diffusion coefficient in the interfacial region, providing microscopic insight into the increase in interfacial resistance. Finally, the method by which LiNbO*_y_* coatings suppress LPSC decomposition was elucidated, providing essential insights into interfacial‐resistance evolution and coating‐thickness design.

## 2 Results

### 2.1 Low‐Dose‐Rate EELS and Electron‐Irradiation Damage of the Sulfide SE

**FIGURE 1:**
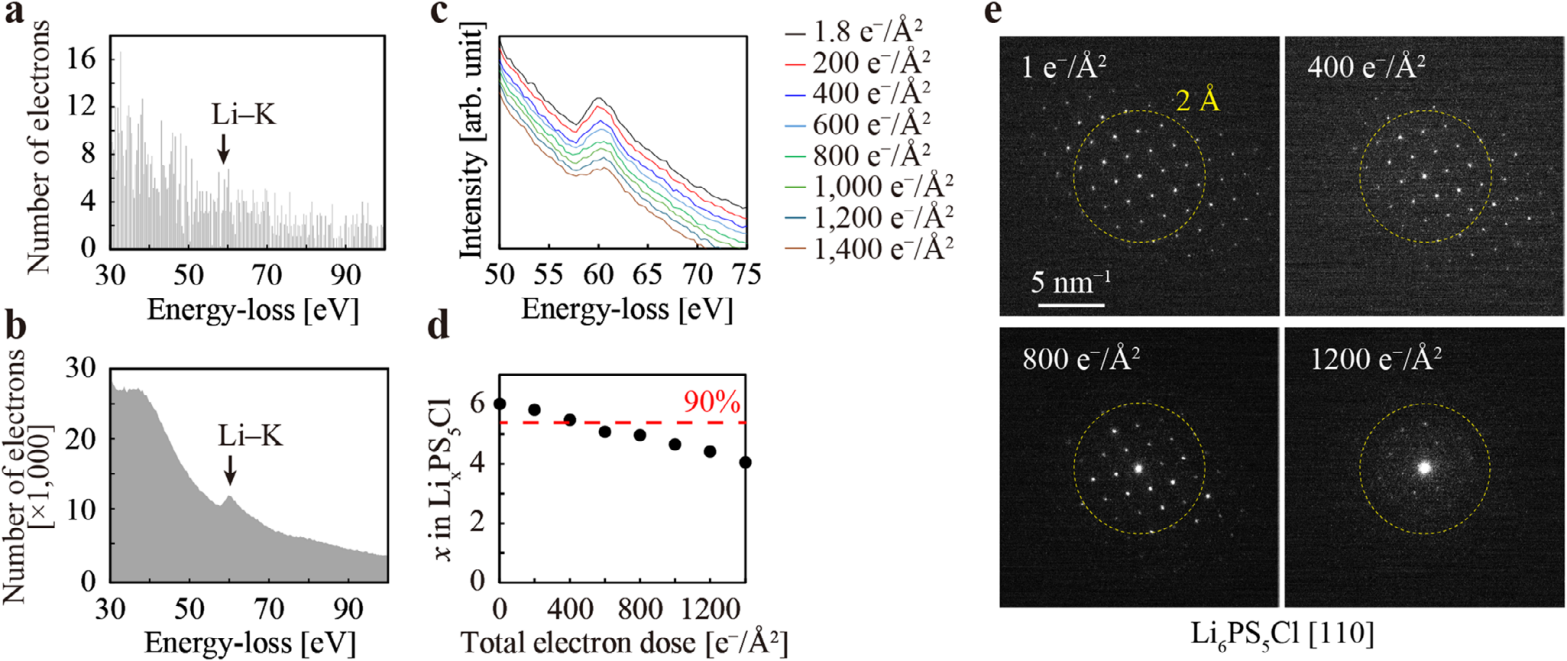
Low‐dose‐rate cryogenic EELS and electron irradiation damage in LPSC. (a) Single‐frame EEL spectrum. (b) Accumulated results of 3,000 EEL spectra. (c) Changes in the Li–K spectra. (d) Change in the Li concentration. Absolute concentrations were determined by normalizing the Li–K edge intensity to a spectrum acquired at an electron dose of 1.8 e^−^ Å^−2^, assuming the stoichiometric composition of Li_6_PS_5_Cl. (e) Change in the electron diffraction pattern acquired along the [110] zone axis of LPSC. The yellow dashed circles indicate the magnitude of the scattering vector corresponding to the P–S nearest‐neighbor distance (∼2 Å), reflecting short‐range structural order in LPSC.

To minimize electron‐beam damage during STEM analysis of the NCM523/LPSC interface, a low‐dose‐rate acquisition scheme was employed in combination with cryogenic conditions and a high accelerating voltage of 300 kV. Because electron‐beam damage in LPSC is predominantly governed by radiolysis, which decreases with increasing accelerating voltage, the use of a high accelerating voltage is essential for mitigating irradiation‐induced degradation. The low‐dose‐rate conditions were established using a single‐crystal LPSC specimen as a model system. Empirical findings indicate that electron irradiation damage in STEM analyses depends not only on the total electron dose but also on the electron‐dose rate. Specifically, a single slow scan at a high probe current can cause greater damage than multiple rapid scans at a lower probe current, even if the total electron doses are identical [39, 40, 41]. Accordingly, a low probe current of ∼3.1 pA and an exposure time of 3.4 × 10^−4^ s pix^−1^ were employed, both significantly lower than those typically used in STEM‐EELS measurements [30]. Consequently, the electron dose rate was reduced by several orders of magnitude compared with conventional STEM‐EELS conditions. However, this significantly low electron dose rate degrades the signal‐to‐noise ratio (SNR) of individual EEL spectra, resulting in inefficient summation because the readout and dark noise become comparable to or larger than the signal. This limitation was mitigated by employing a single‐electron‐counting direct‐detection EELS camera [32], which intrinsically suppresses readout and dark noise. Owing to its intrinsically low noise, successive low‑SNR spectra were integrated over multiple scans, enabling reliable extraction of the underlying signal. Figure 1a shows an EEL spectrum of LPSC that was acquired via a single acquisition with a probe current of 3.1 pA and an exposure time of 3.4 × 10^−4^ s. At approximately 60 eV, where the Li–K edge is located, the intensity is only 1–7 electrons, indicating that the signal is extremely weak and the spectrum is dominated by noise. Figure 1b shows the spectrum obtained by summing 3,000 spectra. The Li–K edge of LPSC is clearly visible, indicating that the Li distribution in LPSC can be captured through multiple scans even under the low‐dose‐rate condition.

Subsequently, the beam tolerance of LPSC under the established low‐dose‐rate condition was evaluated. Because the CEI comprises structurally similar LPSC‐derived components, its beam sensitivity is expected to be of the same order of magnitude as that of the single‐crystal LPSC; therefore, characterizing the bulk material provides a reasonable basis for defining an upper limit for the allowable dose. Figure 1c shows the changes in the Li–K spectra under low‐dose‐rate irradiation at a sample temperature of −150°C. Figure 1d shows the change in the Li concentration in LPSC. When the electron dose is maintained below 400 e^−^ Å^−2^, more than 90% of the original Li concentration can be maintained. Figure 1e shows the changes in the electron diffraction pattern of the single‐crystal LPSC under electron‐beam irradiation. The disappearance of diffraction spots corresponding to *d*‐spacings shorter than ∼2 Å after an electron dose of 800–1,200 e^−^ Å^−2^ suggests that significant disorder develops within the LPSC crystal at this dose. Based on the combined EELS and electron diffraction results, we regarded 400 e^−^ Å^−2^ as a practical upper limit to avoid significant structural degradation of LPSC and therefore limited the total electron dose in all observations to less than this value. Notably, the practical dose limit determined here (400 e^−^ Å^−2^) is approximately two orders of magnitude lower than the doses commonly used for STEM‐EELS analysis of sulfide SEs in the literature (typically > 10^4^ e^−^ Å^−2^) [31]. These findings highlight that sulfide SEs are significantly sensitive to high‐energy electrons.

### 2.2 Potential‐Controlled Sample Preparation

**FIGURE 2:**
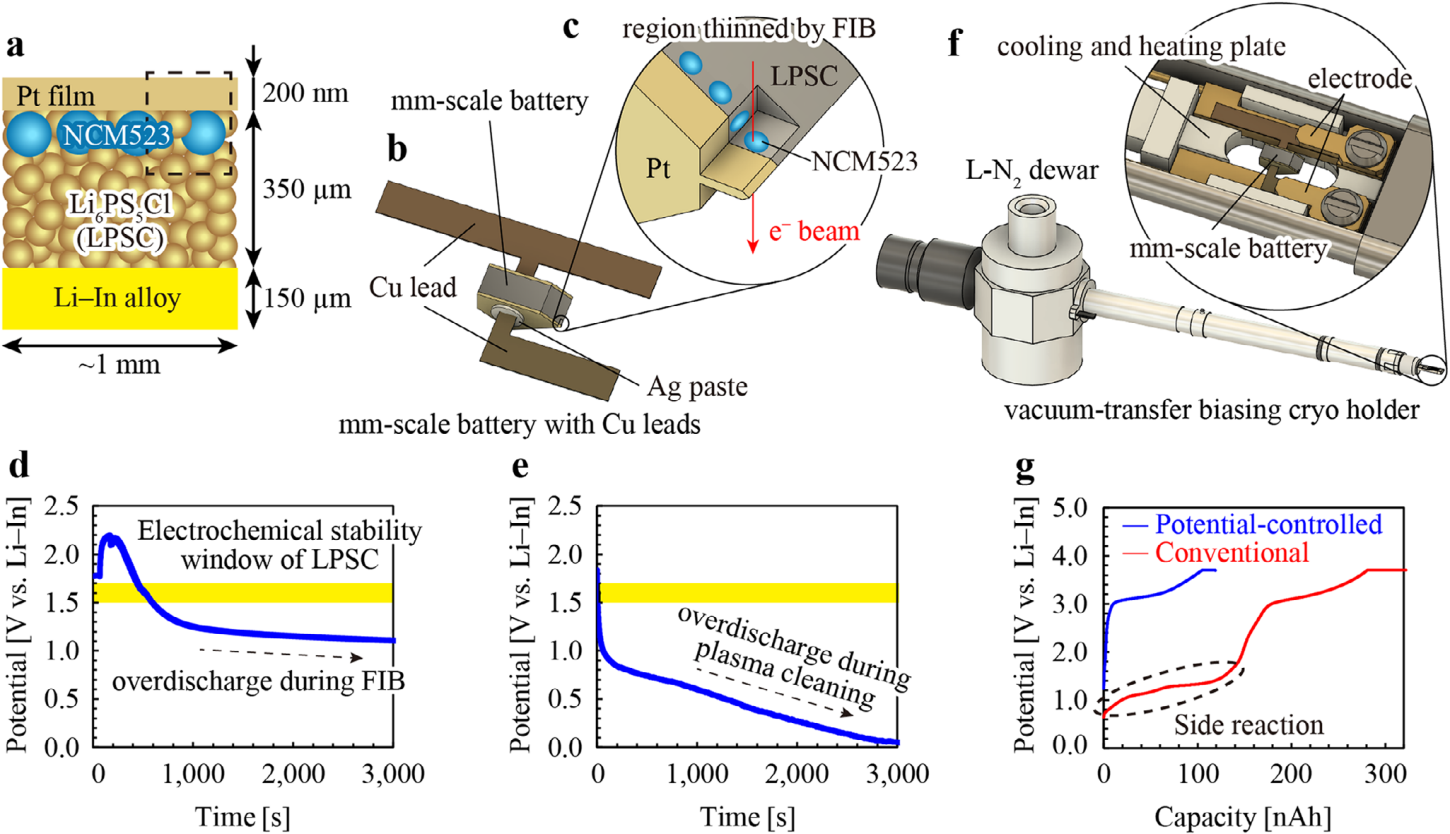
SSB and potential‐controlled sample preparation method. (a) Schematic of the SSB cell. (b) Schematic of a mm‐scale partially sectioned SSB used in this study. (c) Schematic of the region thinned using FIB. (d), (e) Potential variation of the NCM523 working electrode during (d) FIB thinning using a Ga‐ion beam of ∼19 nA and (e) plasma cleaning at ∼330 V. (f) Schematic of a vacuum‐ or inert‐gas‐transfer cryogenic TEM holder equipped with two biasing electrodes and a ceramic heater. (g) Comparison of the first charge curves for samples prepared using the potential‐controlled and conventional (potential‐uncontrolled) methods. The charge measurement was conducted inside a transmission electron microscope at 80°C.

After establishing an effective approach to mitigate electron‐beam damage during observations, the sample‐preparation methodology was considered. Figure 2a shows a schematic of an SSB cell used in this study. Polycrystalline NCM523 particles, which are spherical agglomerates of single crystals with a diameter of a few hundred nanometers, were used as the working electrode. LPSC served as the SE, and a Li–In alloy foil was used as the counter electrode. The cell was fabricated by uniaxial pressing at 120°C. Details of the cell fabrication are described in Note S1. To elucidate the impact of sample preparation on the interphase structure, we employed a mm‐scale SSB with a localized FIB‐sectioned region for TEM observation, as illustrated in Figure 2b. The fabrication procedure was as follows. First, the φ10 mm SSB pellet was cut with a razor blade to obtain small fragments ∼1 mm × 1 mm × t0.5 mm in size. Next, Cu electrodes were attached to the Pt current collector film and Li–In alloy foil of the fragment using solvent‐free Ag paste to ensure reliable electrical contact. The fragment was then mounted on a vacuum‐transfer biasing FIB holder. Subsequently, one edge of the fragment was selectively milled using the FIB to produce an electron‐transparent region that is suitable for TEM observation (Figure 2c). This configuration offers several key advantages for in situ TEM for SSBs compared to conventional micro‐electromechanical systems (MEMS) [42, 43] or probe‐based micro‐batteries [44, 45], whose dimensions are on the order of several to tens of micrometers. First, the larger cell volume (mm‐scale vs. µm‐scale) enables stable electrochemical operation inside a transmission electron microscope, including constant‐current (dis)charging and EIS, which are typically infeasible for the MEMS‐ or probe‐based micro‐battery platforms because their substantially smaller cell volume and capacity prevent precise current control and reliable signal detection. Second, the large SE thickness (∼350 µm, Figure 2a) inherently prevents short circuits, which are known to occur in MEMS‐based micro‐batteries during FIB milling due to sputter redeposition. Such short circuits force the cathode and anode to the same potential, potentially exceeding their stability windows and leading to decomposition of the active materials and interfacial degradation of the SE [46]. This issue must be avoided to ensure that the interphase structure remains unaltered throughout sample preparation. Third, because this method does not require a lift‐out step during FIB processing, the potential difference between the cathode and anode can be continuously monitored and controlled throughout the entire FIB process when using a biasing FIB holder, thereby preventing the unintended potential shift that is caused by Ga‐ion irradiation (details are discussed in the next paragraph). These features make the proposed mm‐scale SSB platform particularly well suited for in situ TEM analysis under conditions that more closely resemble those of practical SSB cells. Despite these advantages, a potential concern with the mm‐scale partially sectioned SSB is that macroscopic electrochemical measurements reflect bulk behavior, whereas STEM analyses probe only the FIB‐thinned region. If the reaction kinetics differ between these two regions, linking TEM observations to macroscopic behavior can result in misleading analyses. However, the operando STEM‐EELS measurements that were conducted in a prior study using an identical configuration confirmed that FIB‐thinned regions (100–200 nm) began to (de)lithiate when voltage was applied, suggesting that thinning does not substantially affect the reaction kinetics [38]. Furthermore, in this study, a 1 h constant‐voltage hold was introduced after each galvanostatic delithiation step to allow Li to redistribute toward equilibrium, thereby ensuring a uniform delithiation state across both the thinned and bulk regions.

Regarding the aforementioned third advantage, to elucidate how Ga‐ion irradiation can influence the electrode potential and interphase structure, the working‐electrode potential was monitored in real time during FIB milling. Figure 2d shows the potential evolution of the NCM523 working electrode during 3,000 s of FIB milling using a Ga‐ion beam current of ∼19 nA. During the initial stage, when the Pt current collector was being sputtered, the potential increased from 1.75 to 2.2 V. As milling progressed into the LPSC region, the potential subsequently decreased to 1.1 V. These observations indicate that sputtering processes and material‐dependent electrostatic charging modulate the local potential distribution within the SSB. This potential imbalance drives unintended charging or discharging currents, which in turn alter the state of charge (SOC) of the SSB. A comparable disturbance was also observed during plasma cleaning, a standard step that is performed after FIB processing to suppress hydrocarbon contamination during STEM observation. As shown in Figure 2e, the working‐electrode potential decreased from 1.83 to 0.05 V during the 3,000 s of plasma cleaning at ∼330 V. Given that the electrochemical stability window of LPSC is ∼1.5–1.7 V (yellow bands in Figure 2d,e) [10], these voltage fluctuations are critical for the interphase structure. To suppress these unintended potential variations, we used a potential‐controlled sample preparation method in which a counter‐balancing current was actively applied to the SSB to maintain the working‐electrode potential at a fixed value throughout both FIB milling and plasma cleaning. The applied compensating currents during these processes are shown in Figure S1 with maximum values of ∼50 and 1,300 nA during FIB milling and plasma cleaning, respectively. Although these currents are small in absolute terms, the limited capacity of the mm‐scale SSB implies that even nA‐level currents can influence its SOC. Under the potential‐controlled protocol, the working‐electrode potential remained unchanged before and after both procedures, confirming that no measurable potential shift occurred during sample preparation.

After thinning and cleaning the cell while maintaining a fixed cell voltage, the SSB was then charged inside a transmission electron microscope using a vacuum‐ or inert‐gas‐transfer cryogenic TEM holder equipped with two biasing electrodes and a ceramic heater, as shown in Figure 2f. Figure 2g shows a comparison of the first charge curves for samples prepared via the potential‐controlled and conventional (potential‐uncontrolled) methods, which were recorded at a constant current of 400 nA and then maintained at a constant voltage of 3.7 V for 1 h. The measurement was conducted at 80°C inside the microscope to accelerate the reaction, thereby driving the interface closer to equilibrium. In the sample prepared using the conventional method, unintended over‐discharge during the FIB and plasma cleaning processes produced an anomalous low‐voltage plateau (side reaction), whereas the potential‐controlled sample displayed the typical charging curve for NCM523 and Li–In. Such a side reaction likely originated from structural or chemical changes in the interphase or in the active material near the interface. This result shows the effectiveness of our proposed potential‐controlled sample preparation method.

### 2.3 In Situ Observation of the Li Depletion and Structural Degradation at the Uncoated NCM523/LPSC Interface

**FIGURE 3:**
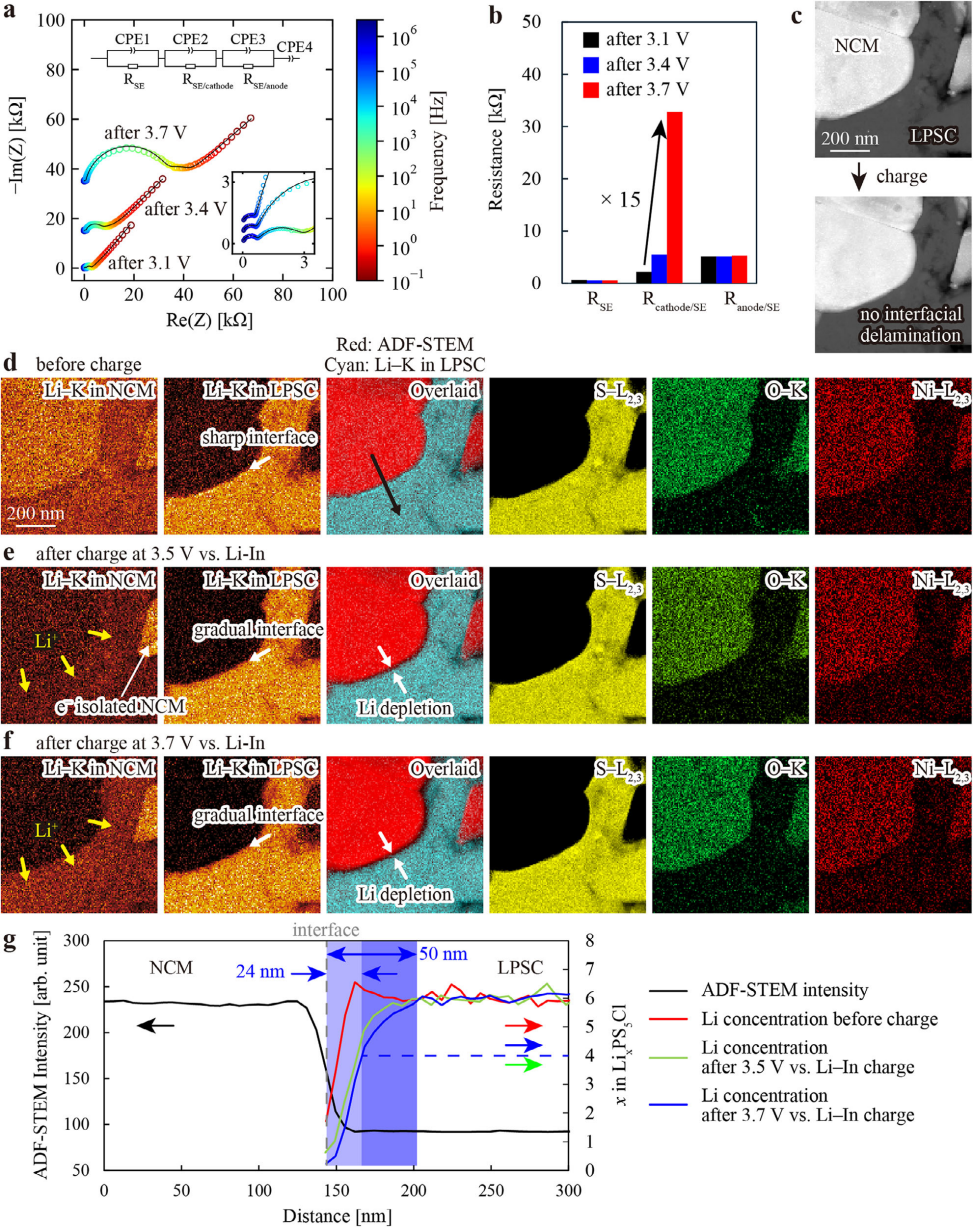
In situ imaging of Li depletion at the uncoated NCM523/LPSC interface. (a) Nyquist plots for a mm‐scale SSB obtained under open‐circuit conditions at 80°C after 1 h floating tests at 3.1, 3.4, and 3.7 V vs. Li–In. The floating tests were performed sequentially using the same cell inside a transmission electron microscope in the order of 3.1, 3.4, and 3.7 V vs. Li–In, with the cell voltage returned to 3.1 V before each EIS measurement. The equivalent circuit used for fitting is shown in the upper inset. Colored symbols represent the experimental EIS data, and the solid black lines correspond to the fitted curves. The lower‐right inset shows an enlarged view of the high‐frequency region. (b) Resistances extracted from the fits, showing their evolution with the applied floating voltage. (c) ADF‐STEM images of the NCM523/LPSC interface observed before and after charging. (d–f) Elemental distributions (d) before charge, (e) after charge at 3.5 V vs. Li–In, and (f) after charge at 3.7 V vs. Li–In. (g) Line profiles of ADF‐STEM intensity and Li concentration near the interface before and after charging. The Li concentration was quantified by normalizing the integrated intensity of the Li–K edge, which is proportional to the local Li content, to that of the bulk LPSC region, assumed to have the stoichiometric composition of Li_6_PS_5_Cl. Possible uncertainties associated with specimen thickness, spectral overlap, plural scattering, and electron‐beam‐induced Li loss are discussed in Supporting Information.

With the SOC and interphase structure preserved, the evolution of interfacial resistance was examined using EIS. Figure 3a,b illustrate the electrochemical impedance response of the mm‐scale SSB after constant‐voltage holding at different potentials for the potential‐controlled sample. Figure 3a shows the Nyquist plots that were acquired after 1 h voltage holds at 3.1, 3.4, and 3.7 V at 80°C. The voltage‐hold steps were performed sequentially using the same cell, and after each voltage hold, the cell voltage was returned to 3.1 V and the EIS spectrum was measured under open‐circuit conditions. The semicircles in the high‐frequency region (> 10^5^ Hz) are attributed to the resistance of the LPSC layer (lower‐right inset), whereas those in the middle‐frequency region (10^5^–10^2^ Hz) correspond to the interfacial charge‐transfer resistance between NCM523 and LPSC [47]. With increasing holding voltage, the overall impedance increased markedly, and a pronounced semicircle was obtained after the 3.7 V hold. The impedance spectra were fitted using the equivalent circuit shown in the upper inset, which separates the contributions from the SE (R_SE_), cathode interface (R_cathode/SE_), and anode interface (R_anode/SE_). Figure 3b shows a comparison of the resistance components extracted from the fitting. While R_SE_ and R_anode/SE_ remain nearly unchanged regardless of the holding voltage, R_cathode/SE_ increases significantly after the 3.7 V hold. The diameter of the semicircle at the middle frequency exhibited a 15‐fold increase compared to that after the 3.1 V hold, from 2,143 to 32,787 Ω, indicating that chemo‐mechanical degradation led to an increase in interfacial resistance. To further assess the origin of the increase in interfacial resistance, the interfacial behavior was evaluated using a conventional pellet‐type solid‐state electrochemical cell, as shown in Figure S2. During the constant‐voltage hold at 3.7 V, the cathode interfacial resistance gradually increased over a period of 1,000 h (Figure S2c) at room temperature. While mechanical degradation, such as interfacial delamination, is expected to contribute to resistance evolution, the continuous increase observed under prolonged voltage holding indicates that electrochemical degradation plays a significant role in the development of interfacial resistance.

To visualize changes in the interphase structure associated with the increase in interfacial resistance, an uncoated NCM523/LPSC interface was observed at the same location before and after charging using in situ cryogenic STEM‐EELS. The charging tests were conducted inside a transmission electron microscope by heating the SSB to 80°C and applying a constant current of 200 nA. After reaching 3.5 or 3.7 V, the SSB was maintained at the constant voltages for 1 h to allow the interface to reach equilibrium. Notably, when evaluated in terms of the increase in interfacial resistance, the floating test at 3.7 V for 1 h at 80°C corresponds to ∼513 h (∼21 days) at 25°C (Figure S2), indicating an approximately 513‐fold acceleration relative to room temperature. After charging at each voltage, the SSB sample was cooled to −150°C for STEM‐EELS observation with minimized electron‐beam‐induced damage. No deformation of the sample was observed upon heating and cooling. Figure 3c shows annular dark‐field (ADF)‐STEM images of the interface between NCM523 and LPSC before and after charge. Low‐magnification ADF‐STEM images are illustrated in Figure S3. Figure 3d–f show the changes in elemental distributions before and after charging to 3.5 and 3.7 V; the images were acquired using the above‐mentioned low‐dose‐rate cryogenic scheme. Each STEM‐EELS dataset was acquired by accumulating 55 scans. The spatial sampling interval and electron dose for a single STEM‐EELS measurement were 6 nm pix^−1^ and 100 e^−^ Å^−2^, respectively. After charging, mechanical delamination was observed at certain interfaces (Figure S4); however, such particles were excluded from the analysis because this study focused on electrochemical degradation. Li maps in NCM523 (first column of panels from the left) show that Li ions were extracted from the NCM523 as charging progressed, confirming that the observed NCM523 particle was indeed charged inside the microscope. The Li concentration of a small particle located at the right edge remained unchanged, which is attributed to the absence of an electronic conduction pathway to the current collector (Figure S3). Li maps in LPSC (second column of panels from the left) show that a sharp interface was present before charging, whereas more gradual interfaces appeared after charging (indicated by white arrows). Note that the Li–K signals from NCM523 and LPSC were distinguished based on differences in their peak positions and shapes (Figure S5). The third column of panels from the left shows overlaid maps of ADF‐STEM (red) and Li distribution in LPSC (cyan). Because the ADF‐STEM intensity is high in the NCM523 region, the red color represents the NCM523 area, whereas the cyan color indicates the Li distribution in LPSC near the interface. After charging to 3.5 and 3.7 V, a dark, band‐like contrast became evident near the interface, which was not present before charging. These observations indicate the formation of a Li‐depleted layer on the LPSC side of the interface. Because no pronounced decrease in Li concentration was detected in regions away from the interface, electron‐beam damage could not have caused this phenomenon. Considering the applied potential and the known oxidative instability of LPSC, this Li depletion is likely associated with electrochemical oxidation of LPSC. Notably, the Li‐depleted layer exhibited a uniform thickness along the interface, rather than localized or domain‐like features. The distributions of S, O, and Ni showed no significant changes. Considering that SO*_x_* and PO*_x_* species have been detected at degraded NCM523/sulfide‐SE interfaces in previous studies using ToF‐SIMS [23, 24], this result may appear somewhat unexpected. Possible explanations include differences in the electrochemical cycling conditions and cell configuration. A detailed discussion is provided in Note S2. A discussion on the electronic state of S is also provided in Note S3. Figure 3g shows the line profiles of the ADF‐STEM intensity and quantitative Li concentrations in LPSC before and after charging along the black line that is indicated in Figure 3d. The NCM523/LPSC interface was defined as the point where the ADF‐STEM intensity was intermediate between those of the NCM523 and LPSC regions. The thickness of the Li‐depleted layer was defined as the distance over which the Li concentration in LPSC recovered to the bulk level (*x* = 6 in Li*_x_* PS_5_Cl). Based on these definitions, a Li‐depleted layer that was approximately 50 nm thick was formed after charging to 3.7 V. Similar Li‐depleted interfacial layers with thicknesses of approximately 50 nm were reproducibly observed at five uncoated NCM523/LPSC interfaces under the same charging conditions. Within 24 nm of the interface, the Li concentration decreased significantly to below *x* = 4 in Li*_x_* PS_5_Cl.

**FIGURE 4:**
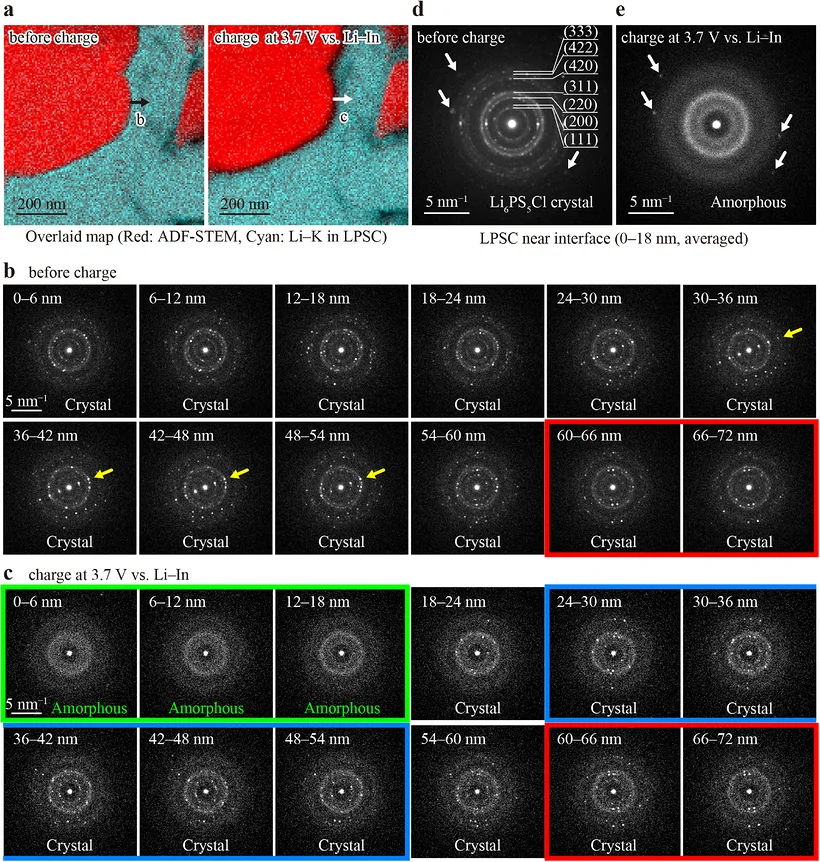
Crystallinity analysis of the Li‐depleted region at the uncoated NCM523/LPSC interface. (a) Overlaid maps of ADF‐STEM (red) and Li distribution in LPSC (cyan) before and after charging at 3.7 V vs. Li–In. (b) EF‐NBD patterns collected from LPSC before charging, exhibiting clear diffraction spots corresponding to crystalline LPSC. (c) EF‐NBD patterns obtained after charging to 3.7 V vs. Li–In, showing that the LPSC region within 0–18 nm of the interface became amorphous. (d,e) Averaged EF‐NBD patterns from the LPSC near the interface (0–18 nm) before and after charging to 3.7 V vs. Li–In, respectively. The white arrows indicate diffraction spots originating from the NCM523 that marginally overlapped into the analyzed region.

To visualize the evolution of the crystal structure within the Li‐depleted interfacial region, EF‐SNBD was employed. The same region examined via STEM‐EELS was observed both before and after charging to 3.7 V. EF‐SNBD employs a nanometer‐scale electron probe with a small convergence semi‐angle that is raster‐scanned across the specimen. A post‐column EEL spectrometer selects only elastically scattered electrons (*E_loss_* = 0 ± 10 eV), thereby removing inelastically scattered background. Consequently, at each probe position, the filtered electrons form a high‐quality 2D electron diffraction pattern on a detector. A comparison of NBD patterns acquired with and without an energy filter is shown in Figure S6. The spatial sampling interval used in EF‐SNBD was 6 nm pix^−1^. The electron doses before and after charging were 25 and 7 e^−^ Å^−2^, respectively. Accordingly, three STEM‐EELS measurements (before charging and after charging to 3.5 and 3.7 V) and two EF‐SNBD measurements (before charging and after charging to 3.7 V) were obtained, resulting in a total electron dose of ∼332 e^−^ Å^−2^ in the analyzed area. This dose was below the degradation threshold of LPSC (400 e^−^ Å^−2^), as illustrated in Figure 1c–e. Figure 4a shows the overlaid maps of ADF‐STEM (red) and Li distribution in LPSC (cyan) before and after charging to 3.7 V. These maps correspond to the third panels from the left in Figure 3d,f. Figure 4b,c show the EF‐NBD patterns obtained from the LPSC region near the interface before and after charging, respectively, along the direction indicated by the arrow in Figure 4a. The EF‐NBD patterns exhibiting distinct diffraction spots are labeled as “Crystal,” whereas those without such spots are labeled as “Amorphous.” Before charging, diffraction spots and Debye–Scherrer rings were observed throughout the region extending from 0 to 72 nm away from the NCM523/LPSC interface, indicating that the LPSC was crystalline irrespective of the distance from the interface. Figure 4d shows the averaged EF‐NBD pattern near the interface (0–18 nm) before charging. The Debye–Scherrer rings can be indexed to the LPSC crystal (space group: $F\bar{4}3m$). In contrast, Figure 4c shows that, after charging to 3.7 V, diffraction spots disappeared, and diffuse halo rings appeared near the interface (0–18 nm), as indicated by the green rectangle. Figure 4e shows the averaged EF‐NBD patterns near the interface (0–18 nm) after charging to 3.7 V. The spatial extent of the amorphous region (0–18 nm) closely corresponds to that of the Li‐depleted region (*x* < 4 in Li*_x_* PS_5_Cl, 0–24 nm) that was identified by STEM‐EELS, confirming a correlation between Li depletion and the degradation of crystallinity. These results demonstrate that LPSC became amorphous in the strongly Li‐depleted region (*x* < 4). This structural degradation is likely associated with electrochemical oxidation of LPSC under the applied charging conditions. The LPSC located more than 24 nm from the interface retained distinct diffraction spots, indicating that the electrolyte remained crystalline under these charging conditions. However, in the region 24–54 nm away from the interface (sky blue rectangle in Figure 4c), multiple diffraction spots (such as the systematic row of reflections indicated by yellow arrows in Figure 4b) disappeared. This result indicates that, although the LPSC in the 24–54 nm region remained crystalline, its crystal structure had begun to degrade. The degraded crystalline region (24–54 nm) spatially corresponds to the Li‐depleted region (4 < *x* < 6 in Li*_x_* PS_5_Cl, 24–50 nm) identified by STEM‐EELS, further supporting the close correlation between Li depletion and structural degradation. In the region that was more than 60 nm away from the interface, the EF‐NBD patterns remained nearly unchanged before and after charging, as shown by the red rectangles in Figure 4b,c, indicating that the interfacial reaction did not affect this area. Because LPSC is a crystalline Li‐ion conductor, the decrease in carrier‐ion concentration and blockage of ion‐conduction pathways in the LPSC lattice likely reduced the Li‐ion conductivity within the Li‐depleted and structurally degraded interfacial region, thereby leading to an increase in interfacial resistance, as shown in Figure 3a,b.

### 2.4 Suppression of Interfacial Li Depletion and Structural Degradation by LiNbO*_y_* Coating

**FIGURE 5:**
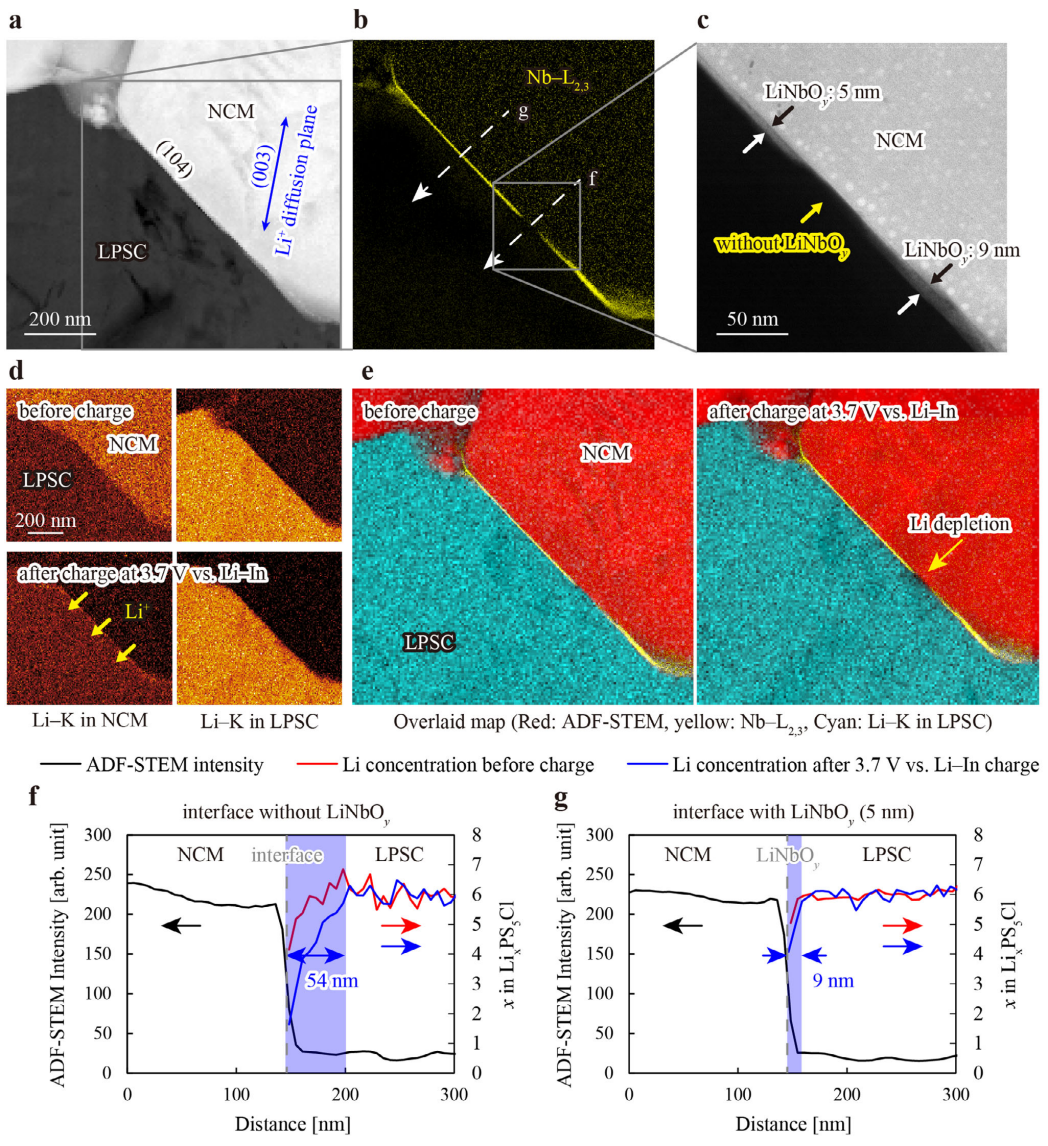
In situ imaging of Li depletion at the LiNbO*_y_*‐coated NCM523/LPSC interface. (a) ADF‐STEM image. (b) Integrated intensity distribution of Nb–L_2,3_ in the region indicated by the gray rectangle in (a). (c) High‐magnification ADF‐STEM image of the region indicated by the gray rectangle in (b). (d) Li maps in NCM523 and LPSC before and after charging to 3.7 V vs. Li–In. (e) Overlaid maps of ADF‐STEM (red), Nb distribution (yellow), and Li distribution in LPSC (cyan) before and after charging. (f,g) Line profiles of ADF‐STEM intensity and Li concentration in LPSC before and after charging at the uncoated and LiNbO*_y_*‐coated interfaces, respectively.

**FIGURE 6:**
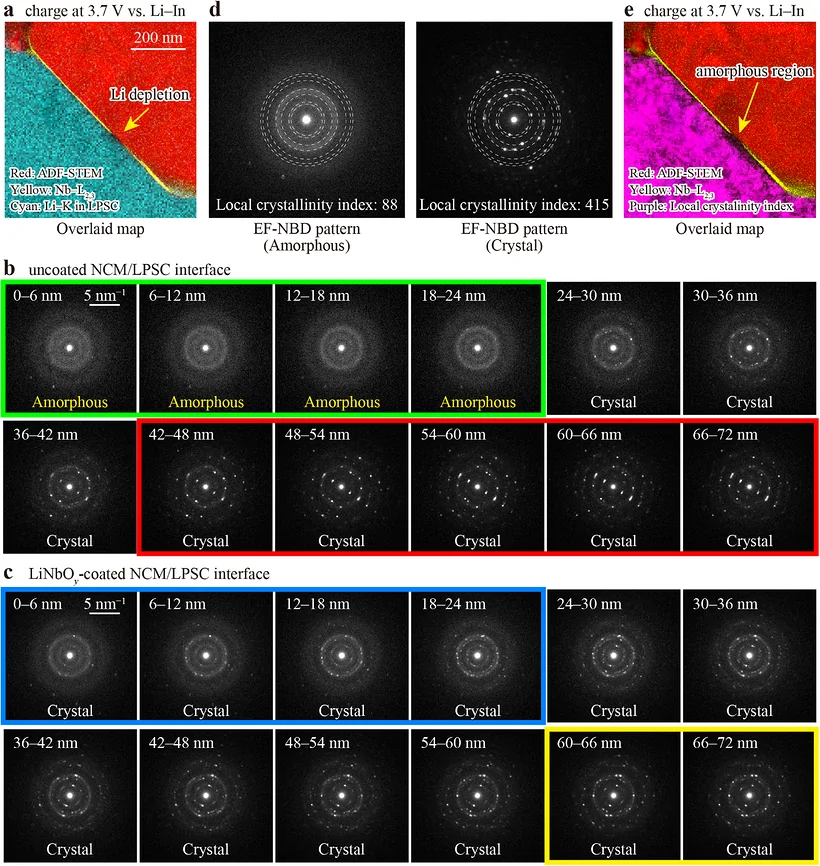
Crystallinity analysis at the LiNbO*_y_*‐coated NCM523/LPSC interface after charging to 3.7 V vs. Li–In. (a) Overlaid maps of ADF‐STEM (red), Nb distribution (yellow), and Li distribution in LPSC (cyan). (b) EF‐NBD patterns collected near the interface between the uncoated NCM523 and LPSC along the dashed arrow that is shown in Figure 5b. (c) EF‐NBD patterns collected near the interface between the LiNbO*_y_*‐coated (5 nm thick) NCM523 and LPSC along the dashed arrow that is shown in Figure 5b. (d) EF‐NBD patterns from LPSC near and away from the uncoated interface. The dashed circles indicate the region where diffraction spots from LPSC appear (refer to Figure 4d). (e) Overlaid maps of ADF‐STEM (red), Nb distribution (yellow), and local crystallinity (*LC*) index (purple). The *LC* index represents the difference between the maximum and minimum diffraction intensities along the dashed circles in (d). Regions with low crystallinity appear dark purple, whereas those with high crystallinity appear bright purple.

To examine the effect of the LiNbO*_y_* coating, the interface between LiNbO*_y_*‐coated NCM523 (average coating thickness: 9 nm) and LPSC was analyzed. Figure 5a shows an ADF‐STEM image of the region near the LiNbO*_y_*‐coated NCM523/LPSC interface. Low‐magnification ADF‐STEM images are shown in Figure S7. In Figure 5a, the NCM523 (104) plane is in contact with LPSC at the interface. Because the (104) plane is crystallographically stable in NCM523, the interface was considered to be flat. The Li conduction plane (003) is indicated by the double‐headed blue arrow. Figure 5b shows the integrated intensity distribution of the Nb–L_2,3_ edge acquired via EELS in the region indicated by the gray rectangle in Figure 5a. Note that the Nb–L_2,3_ edge is located at approximately 2,400 eV, which requires intense electron irradiation that can cause severe beam damage to LPSC. Therefore, the Nb distribution was acquired at the end of the measurements, after the in situ STEM‐EELS (Figure 5d,e) and EF‐SNBD analyses (Figure 6b–e). The NCM523 surface was predominantly covered with LiNbO*_y_*; however, a part of the surface remained uncoated. Figure 5c shows a high‐magnification ADF‐STEM image of the region indicated by the gray rectangle in Figure 5b. Because electron‐beam damage was a concern at this magnification, this image was also acquired at the end, after the in situ STEM‐EELS and EF‐SNBD analyses. The interfacial LiNbO*_y_* layer can be clearly observed, with thicknesses of ∼5 and 9 nm in the upper‐left and lower‐right regions, respectively. In the center of the image, an exposed NCM523 surface without LiNbO*_y_* is visible. Figure 5d shows the Li maps in NCM523 and LPSC before and after charging to 3.7 V. The distributions of other elements (S, O, and Ni) are shown in Figure S8. The charging condition was identical to that used for the experiment shown in Figure 3, that is, a floating test at 80°C for 1 h at 3.7 V. The STEM‐EELS acquisition condition was also identical to that used to obtain Figure 3d–f, that is, an electron dose of 100 e^−^ Å^−2^ per STEM‐EELS acquisition. Compared to the NCM523 sample without the LiNbO*_y_* coating, the increase in the interfacial resistance was limited to a factor of 2.3 (Figure S9). Similar to the Li maps in NCM523 shown in Figure 3d–f, Li extraction from NCM523 can be observed in Figure 5d, confirming that the observed particle was charged inside the microscope. Figure 5e shows overlaid maps of ADF‐STEM (red), Nb distribution (yellow), and Li distribution in LPSC (cyan) before and after charging to 3.7 V. After charging, a dark contrast is evident near the interface with the uncoated NCM523 surface, as indicated by the yellow arrow, whereas no contrast change is observed at the LiNbO*_y_*‐coated interfaces. Figure 5f,g show the line profiles of the ADF‐STEM intensity and quantitative Li concentration in LPSC along the dashed white arrows that are shown in Figure 5b, for the uncoated and LiNbO*_y_*‐coated interfaces, respectively. At the interface without LiNbO*_y_* coating, a 54‐nm‐thick Li‐depleted region was formed upon charging, which is consistent with the thickness observed in Figure 3g. Conversely, no Li‐depleted layer was observed at the LiNbO*_y_*‐coated interface, even with a coating thickness of 5 nm. Similar suppression of pronounced Li depletion was reproducibly observed at three LiNbO*_y_*‐coated NCM523/LPSC interfaces. These results indicate that, within the coating‐thickness range investigated in this study, a LiNbO*_y_* layer as thin as approximately 5 nm can suppress interfacial Li depletion. Because interfaces with coating thicknesses below 5 nm were not examined, this value should not be regarded as the minimum effective coating thickness. Within the investigated thickness range, coating coverage appears to be more important than any further increase in the local coating thickness. A recent study reported approximately 2.5 nm as the minimum effective thickness of a LiNbO*_y_* coating for suppressing interfacial side reactions in sulfide‐based SSBs [21]. Our observation is consistent with this report and does not exclude the effectiveness of thinner coatings.

To investigate the crystal structure at the LiNbO*_y_*‐coated interface, EF‐SNBD analysis was performed. Figure 6a shows the overlaid maps of ADF‐STEM (red), Nb distribution (yellow), and Li distribution in LPSC (cyan) after charging to 3.7 V, which is identical to the panel on the right of Figure 5e. Figure 6b,c show the EF‐NBD patterns obtained from the LPSC regions near the uncoated and LiNbO*_y_*‐coated interfaces, respectively. The EF‐NBD patterns were acquired after charging, along the direction indicated by the white dashed arrows in Figure 5b. The data obtained before charging are shown in Figure S10. The spatial sampling interval of EF‐SNBD and electron dose for a single EF‐SNBD acquisition were 6 nm pix^−1^ and 25 e^−^ Å^−2^, respectively. Accordingly, two STEM‐EELS and EF‐SNBD measurements each (before and after charging to 3.7 V) were obtained, resulting in a total electron dose of ∼250 e^−^ Å^−2^ in the analyzed area. This value was below the degradation threshold of LPSC (400 e^−^ Å^−2^), as discussed in Figure 1c–e. Diffuse halo rings, instead of distinct diffraction spots, were observed in the interfacial region (0–24 nm) of the uncoated interface, as marked by the green rectangle in Figure 6b. This finding reveals that the crystalline LPSC that was adjacent to the NCM523 surface became amorphous upon charging, which is consistent with the results shown in Figure 4. In the region located more than 42 nm away from the interface (red rectangle in Figure 6b), the EF‐NBD patterns exhibited well‐defined diffraction spots, indicating that the charging‐induced amorphization and structural degradation were confined to the interfacial region. In contrast, the EF‐NBD pattern obtained from the LiNbO*_y_*‐coated interface (sky‐blue‐colored rectangle in Figure 6c) exhibited distinct diffraction spots. This indicates that the LiNbO*_y_* coating suppressed the complete amorphization of LPSC near the interface, which is consistent with the Li distribution analysis shown in Figure 5g. However, compared to the region that was more than 60 nm away from the interface (yellow rectangle in Figure 6c), the overlapping halo‐ring intensity was marginally stronger in the interfacial region (0–24 nm, sky‐blue rectangle), suggesting the onset of structural degradation; nevertheless, the extent of degradation was suppressed compared to that observed at the uncoated interface.

Next, to map the spatial distribution of amorphous and crystalline regions within LPSC, a *local crystallinity (LC)* index was introduced. The *LC* index was defined as the difference between the maximum and minimum diffraction intensities measured along the radial distances corresponding to the (111), (200), (220), (311), (420), (422), and (333) reflections of LPSC. Figure 6d shows representative EF‐NBD patterns of amorphous and crystalline regions. The dashed circles in the patterns correspond to these reflections, as shown in Figure 4d. In the amorphous region, only diffuse halo rings are observed, and the diffraction intensity along each dashed circle is nearly uniform, resulting in a small *LC* index. In contrast, in the crystalline region, diffraction spots produce large intensity fluctuations along the dashed circles, leading to a large *LC* index. Figure 6e shows the overlaid maps of ADF‐STEM intensity (red), Nb distribution (yellow), and *LC* index (purple) after charging to 3.7 V. The bright‐ and dark‐purple regions represent highly and poorly crystalline regions, respectively. As indicated by the yellow arrow, a region with a low *LC* index is observed near the uncoated NCM523/LPSC interface. This distribution correlated well with the Li‐depleted layer that is shown in Figure 6a, demonstrating a strong correlation between Li concentration and crystal structure. Notably, several regions with a low *LC* index were scattered within the bulk LPSC. These regions correspond to LPSC areas oriented such that diffraction spots are less likely to appear. The EF‐NBD patterns from such regions are shown in Figure S11. An amorphous halo pattern was observed only near the uncoated NCM523/LPSC interface.

### 2.5 Machine‐Learning‐Potential Molecular Dynamics Simulations of Li Transport in Li‐Depleted LPSC

**FIGURE 7:**
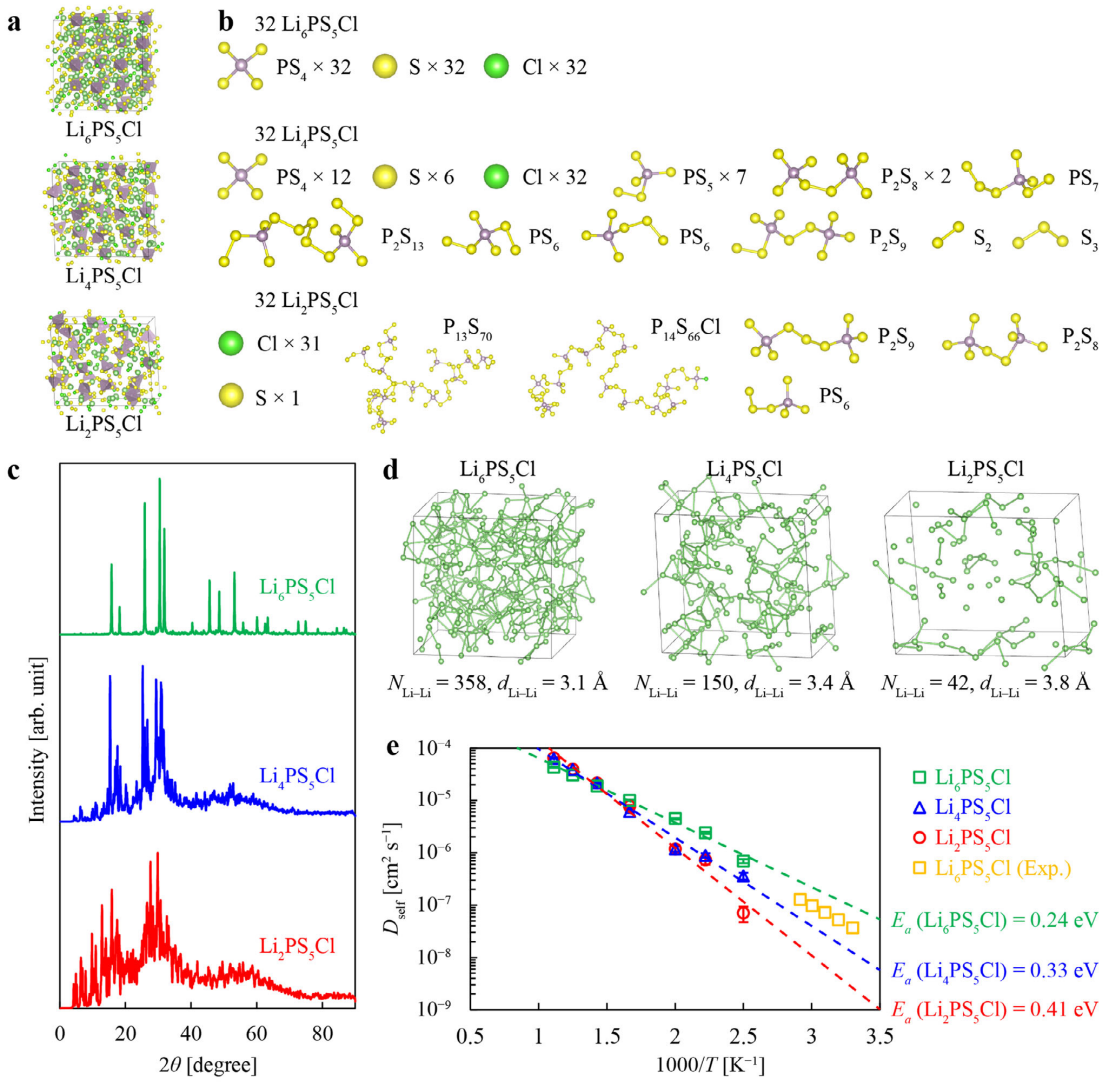
Machine‐learning‐potential MD simulations of Li transport in the Li‐depleted LPSC. (a) Supercell structures obtained after MD simulations at 400 K for Li_6_PS_5_Cl, Li_4_PS_5_Cl, and Li_2_PS_5_Cl. (b) Chemical species in the corresponding MD‐relaxed structures. (c) Simulated X‐ray diffraction patterns of Li_6_PS_5_Cl, Li_4_PS_5_Cl, and Li_2_PS_5_Cl. (d) Qualitative visualization of the Li sublattice using Li–Li connectivity networks constructed using a 3.5 Å cutoff distance to identify nearest‐neighbor Li connections. (e) Arrhenius plots of Li self‐diffusion coefficients (*D*_self_) extracted from MD trajectories using the Einstein relation. Experimental data of pulsed‐field gradient nuclear magnetic resonance (PFG‐NMR) are shown for comparison [54].

To understand how Li depletion affects the structural stability and ionic transport properties, we performed atomistic simulations by systematically removing Li atoms from crystalline Li_6_PS_5_Cl [48]. A 2 × 2 × 2 supercell of Li_6_PS_5_Cl (32 formula units, 192 Li atoms) was constructed as the reference bulk structure. To model the Li‐depleted LPSC observed near the interface, Li atoms were randomly removed from the supercell. Two levels of Li deficiency were considered: Li_4_PS_5_Cl (64 Li atoms removed per supercell) and Li_2_PS_5_Cl (128 Li atoms removed per supercell). These compositions were selected based on the experimentally measured Li‐concentration profile in the interfacial LPSC region (Figure 3g). For each composition, 200 randomized configurations were generated to account for configurational disorder. Each configuration was subjected to geometry and cell optimization using a universal machine‐learning interatomic potential [49, 50, 51]. The lowest‐energy structure among the 200 relaxed configurations was selected for subsequent molecular dynamics (MD) simulations. In these machine‐learning‐potential simulations, electronic degrees of freedom were not treated explicitly. However, neutral Li atoms were removed while maintaining an overall charge‐neutral simulation cell. Thus, the construction of Li_4_PS_5_Cl and Li_2_PS_5_Cl corresponds to the coupled removal of Li^+^ and the associated electron, consistent with the overall oxidation process accompanying electrochemical Li extraction. Because the machine‐learning potential was trained on density functional theory (DFT) energies and forces, changes in the effective interatomic interactions associated with electronic redistribution upon Li removal are represented implicitly to the extent captured by the underlying DFT data. The mean absolute errors of energy and force between the machine‐learning potential and DFT were less than 0.072 eV/atom and 0.051 eV Å^−1^, respectively, for both pristine and Li‐depleted Li*_x_* PS_5_Cl (*x* = 6, 4, 2) models (Figure S12) [49, 52, 53]. These results confirm the computational accuracy of the present potential for the current analysis. MD simulations were performed to evaluate the structural stability and Li transport behavior. The simulations were conducted in the temperature range of 400–900 K with a time step of 1 fs. The representative atomic configurations obtained after the MD simulations at 400 K are shown in Figure 7a. Figure 7b summarizes the chemical species identified in the structures obtained after the MD simulations at 400 K. The fully lithiated Li_6_PS_5_Cl retained its characteristic argyrodite framework throughout the simulations. The isolated PS_4_ tetrahedra were preserved, and the long‐range translational symmetry remained intact. In contrast, Li removal triggered substantial reorganization of the thiophosphate framework. In Li_4_PS_5_Cl, partial disruption of the isolated PS_4_ tetrahedra was observed. S atoms began to form bridging configurations between neighboring P centers, forming short P–(S)*_n_*–P linkages and small polymeric clusters. The transformation became more pronounced in Li_2_PS_5_Cl. Most of the PS_4_ tetrahedra were disrupted, and an extended P–(S)*_n_*–P network developed through S bridging, yielding a polymerized thiophosphate framework rather than the original argyrodite lattice. These results indicate that the amorphization induced by Li removal is not simply a loss of long‐range crystallinity, but is accompanied by substantial chemical reconstruction of the thiophosphate framework. The resulting P–(S)*_n_*–P and other S‐rich bonding motifs are consistent with the local bonding environments of P_2_S*_x_*/P_2_S_5_, polysulfide‐like species, and elemental‐sulfur‐like configurations that have been reported previously as oxidative decomposition products of Li_6_PS_5_Cl [12]. To verify this, we performed DFT optimizations of the Li*_x_* PS_5_Cl (*x* = 6, 4, 2) unit cell. The most stable structures at *x* = 4, 2 in the convex hull, shown in Figure S13, confirm the formation of the P–(S)*_n_*–P network in the Li*_x_* PS_5_Cl, validating the neural network potential calculations. The simulated powder diffraction patterns at 400 K (Figure 7c) corroborate this structural evolution, exhibiting the disappearance of sharp Bragg reflections and the emergence of broad diffuse features, consistent with the loss of long‐range translational symmetry and the onset of amorphization. These features are consistent with experimental observations of amorphization in the Li‐depleted interfacial region, indicating that the simulations successfully reproduce the essential structural evolution upon Li depletion. Notably, this structural collapse occurred within ∼700 ps at 400 K, demonstrating that substantial Li deficiency inherently destabilizes the crystalline LPSC framework. These results establish that Li atoms are not merely mobile charge carriers but integral structural components that maintain the connectivity of the anion framework.

Given that Li transport in argyrodite‐type electrolytes relies on a percolating Li sublattice, we examined the effect of Li depletion on the connectivity of Li diffusion pathways. To qualitatively visualize how the Li sublattice evolves upon Li depletion, Figure 7d presents Li–Li networks constructed by connecting Li ions separated by less than 3.5 Å, a cutoff distance chosen to represent the nearest‐neighbor Li connectivity in argyrodite structures. In Li_6_PS_5_Cl, the Li sublattice forms a highly interconnected 3D network. Upon partial Li removal, the network becomes progressively sparse and less interconnected in Li_4_PS_5_Cl and Li_2_PS_5_Cl. Because the number of Li–Li connections (*N_Li_*_–_ *_Li_*) inherently decreases and the nearest‐neighbor Li–Li distance (*d_Li_*_–_ *_Li_*) increases with decreasing Li concentration, this analysis is intended as a qualitative visualization of the Li sublattice rather than as independent evidence for reduced Li transport. The effect of Li depletion on Li transport was therefore evaluated directly from the MD trajectories through the Li self‐diffusion coefficients calculated from the mean‐squared displacements (MSDs). The corresponding MSD profiles are provided in Figure S14. As shown in the Arrhenius plots (Figure 7e), the extrapolated diffusion coefficient of Li_6_PS_5_Cl at 303 K is 9.4 × 10^−8^ cm^2^ s^−1^, which agrees reasonably with the experimental value of 3.7 × 10^−8^ cm^2^ s^−1^. The calculated activation energy (0.24 eV) is also close to the experimental value of 0.28 eV. The calculated Li self‐diffusion coefficients decrease systematically with Li depletion. The activation energies derived from Arrhenius fits are 0.24, 0.33, and 0.41 eV for Li_6_PS_5_Cl, Li_4_PS_5_Cl, and Li_2_PS_5_Cl, respectively. These dynamical results provide direct evidence that Li migration becomes increasingly hindered with decreasing Li content. The progressive increase in the activation energy with Li removal indicates that, in addition to reducing the charge carrier concentration, Li depletion fundamentally alters the migration energetics. Consequently, the Li‐depleted interfacial region exhibits a significantly lower Li diffusivity than bulk LPSC. The Li‐depleted and structurally degraded LPSC is therefore expected to act as a kinetic bottleneck for Li migration. The reduced Li mobility, caused by Li depletion and the accompanying framework reconstruction, likely contributes to the experimentally observed increase in interfacial resistance. However, the EIS‐derived interfacial resistance also includes contributions from mechanical degradation, which cannot be quantitatively separated in the present study.

## 3 Discussion

**FIGURE 8:**
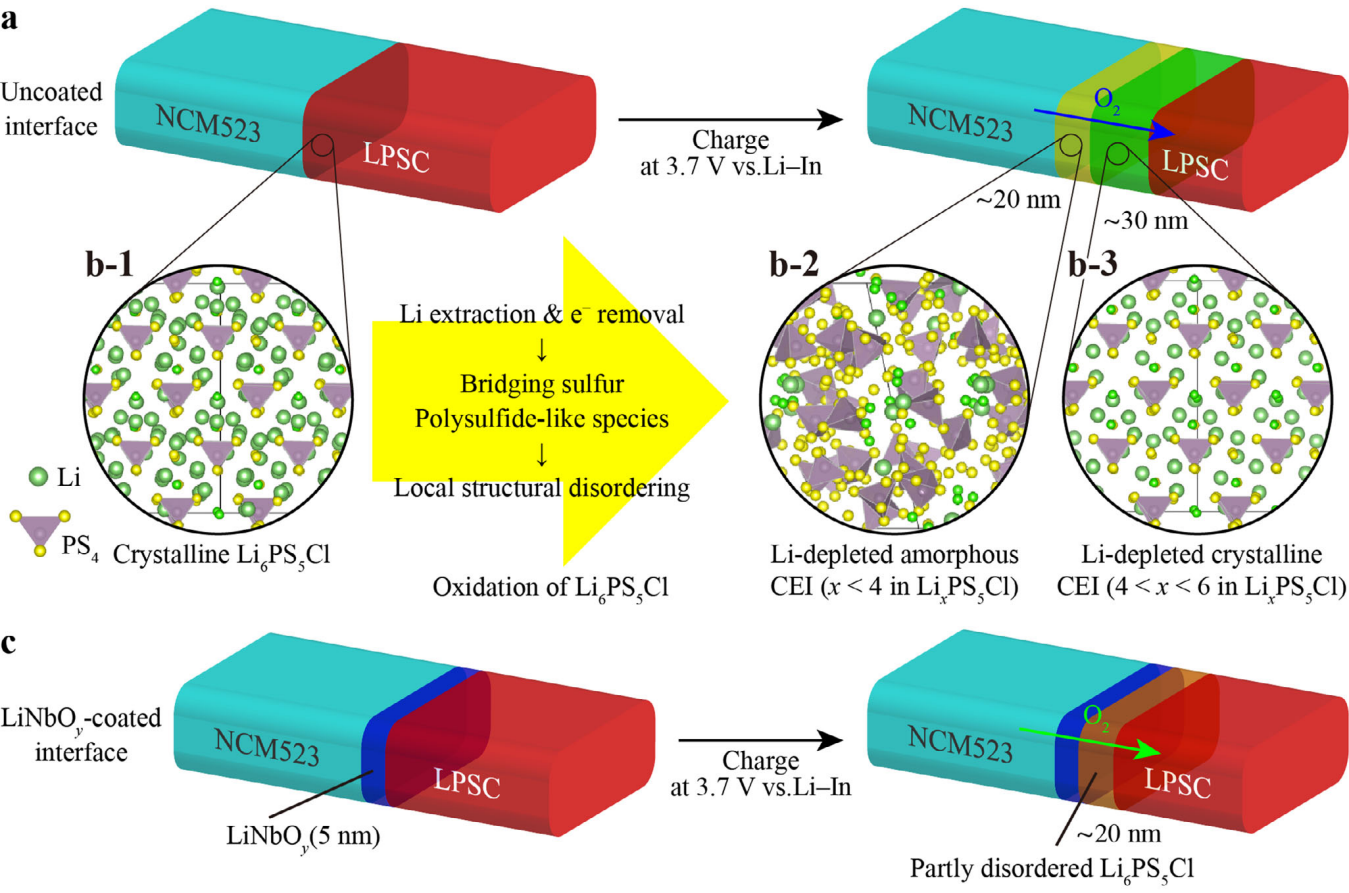
Schematic illustration of the proposed interfacial evolution at uncoated and LiNbO*_y_*‐coated NCM523/LPSC interfaces. (a) Uncoated NCM523/LPSC interface before and after charging to 3.7 V vs. Li–In. (b) Magnified conceptual views of the LPSC near the interface: (b‐1) pristine crystalline LPSC, (b‐2) strongly Li‐depleted amorphized region, and (b‐3) moderately Li‐depleted crystalline region. The Li‐depleted amorphous region is depicted as a chemically reconstructed phase containing P–(S)*_n_*–P and S‐rich bonding motifs suggested by the MD simulations. (c) LiNbO*_y_*‐coated NCM523/LPSC interface before and after charging to 3.7 V vs. Li–In.

Figure 8 schematically illustrates the elemental and structural evolution at the NCM523/LPSC interface during charging, comparing the cases with and without LiNbO*_y_* coating. Figure 8a illustrates the uncoated interface before and after charging. Before charging, NCM523 and LPSC maintain their crystalline structures. The Li concentration in LPSC near the interface is nearly identical to that in the bulk LPSC. Upon charging to 3.7 V, significant changes occur near the interface. An approximately 50‐nm‐thick Li‐depleted interfacial region develops within the LPSC, accompanied by pronounced structural degradation. The region adjacent to NCM523 (∼20 nm thick) transforms into a Li‐depleted amorphous phase (*x* < 4 in Li*_x_* PS_5_Cl). In the region farther from the interface (∼20–50 nm from the interface), LPSC remains crystalline but exhibits marginal structural degradation while becoming deficient in Li (4 < *x* < 6 in Li*_x_* PS_5_Cl). When considered together with the applied electrochemical potential and the known oxidative instability of LPSC, the spatially correlated Li depletion and structural degradation observed near the interface are consistent with CEI formation through electrochemical oxidation of LPSC. Figure 8b illustrates magnified conceptual views of the LPSC near the interface. When the LPSC crystal (Figure 8b‐1) is exposed to high potential beyond its electrochemical stability window, Li ions are extracted from the lattice, and electrons are withdrawn from the S 3p orbitals, leading to oxidation reactions. As electrons are withdrawn, the PS_4_ tetrahedral framework undergoes polymerization, forming –P–S–S–P– and –S–S– containing species. In the region closer to the interface (Figure 8b‐2), both Li and electron depletion occur extensively, leading to the collapse of the periodic PS_4_ network and resulting in complete amorphization. Thus, the amorphous CEI is interpreted as a chemically reconstructed phase characterized by substantial Li depletion and decomposition‐related local bonding motifs, rather than simply as crystalline LPSC that has lost long‐range order. The decrease in carrier concentration and blockage of Li‐ion conduction pathways in the LPSC lattice cause a sharp reduction in ionic conductivity, leading to an increase in interfacial resistance. In the region that is marginally farther from the interface (20–50 nm from the interface, Figure 8b‐3), the extent of Li and electron depletion is smaller, allowing the crystalline structure to be preserved. Figure 8c shows the corresponding states for the LiNbO*_y_*‐coated interface. When a LiNbO*_y_* interlayer is introduced between NCM523 and LPSC, neither pronounced Li depletion nor complete amorphization is observed after charging. Only marginal structural modification occurs within ∼20 nm of the interface, indicating that the LiNbO*_y_* coating suppresses interfacial decomposition and preserves the crystallinity of the SE. Overall, the comparison highlights the crucial role of the LiNbO*_y_* coating in preventing Li loss and structural collapse of LPSC near the interface, thereby stabilizing the electrochemical and structural integrity of the SSB interface. Although the LiNbO*_y_* coating suppresses direct contact between NCM523 and LPSC, thereby limiting direct electrochemical degradation of LPSC, oxygen‐related degradation may also contribute to the interfacial reaction, particularly at high states of charge. Oxygen released from highly charged NCM523 may migrate toward the sulfide electrolyte and promote the formation of oxygen‐containing degradation products such as PO*_x_* and SO*_x_*. Such reactions may contribute to the remaining interfacial resistance even at LiNbO*_y_*‐coated interfaces. Quantitative separation of the contributions of ionic transport degradation within the interphase, mechanical processes such as interfacial delamination (Figure S4), and possible oxygen‐mediated degradation remains an important subject for investigation in future work.

## 4 Conclusion

In this study, the electrochemical degradation and structural evolution at the NCM523/LPSC interface were directly visualized using in situ STEM‐EELS and EF‐SNBD. To enable nanoscale observations without inducing artificial degradation, a potential‐controlled protocol was developed to preserve the native interphase structure during sample preparation processes such as FIB thinning and plasma cleaning. Moreover, a combination of low‐dose‐rate acquisition and cryogenic techniques was employed to suppress electron‐beam‐induced damage during STEM observation. Through systematic low‐dose experiments, the degradation threshold of the sulfide SE was determined, enabling high‐resolution interfacial imaging with a spatial resolution of 6 nm pix^−1^ while minimizing irradiation‐induced damage. By comparing interfaces with and without LiNbO*_y_* coatings via in situ observations, the structural and electrochemical roles of the coating layer during charging were elucidated. At the uncoated NCM523/LPSC interface, charging to 3.7 V resulted in the formation of a ∼50‐nm‐thick Li‐depleted CEI. Within ∼20 nm of the interface, pronounced Li depletion (*x* < 4 in Li*_x_* PS_5_Cl) occurred, accompanied by amorphization of LPSC. In the region located farther from the interface (∼20–50 nm away), LPSC retained its crystalline structure with only slight structural degradation, while showing moderate Li depletion (4 < *x* < 6 in Li*_x_* PS_5_Cl). In contrast, neither pronounced Li depletion nor complete amorphization was observed at the LiNbO*_y_*‐coated interface, demonstrating that the coating suppresses interfacial degradation. Within the coating‐thickness range investigated in this study, a LiNbO*_y_* layer as thin as approximately 5 nm suppressed the formation of the Li‐depleted CEI, suggesting that coating coverage may be more important than further increasing the local coating thickness for minimizing interfacial resistance. These findings provide direct nanoscale evidence that the LiNbO*_y_* coating functions as a buffer layer, preserving the ionic concentration and crystallinity of the SE and stabilizing the interface. Complementary machine‐learning‐potential MD simulations revealed that Li depletion destabilizes the Li_6_PS_5_Cl framework, causing amorphization and reduced Li mobility. The calculated activation energy for Li self‐diffusion increased from 0.24 eV for Li_6_PS_5_Cl to 0.33 and 0.41 eV for Li_4_PS_5_Cl and Li_2_PS_5_Cl, respectively, indicating that the Li‐depleted CEI acts as a kinetic bottleneck that increases the interfacial resistance. The experimental framework established here offers a versatile methodology that can also be applied to anode/SE interfaces, thereby enabling the derivation of interfacial design principles for SSBs.

## Author Contributions

**Yuki Nomura**: conceptualization, methodology, investigation, visualization, funding acquisition, writing – original draft, formal analysis, data curation. **Ryoma Sasaki**: methodology, investigation, writing – review and editing, software, formal analysis. **Huu Duc Luong**: methodology, investigation, writing – review and editing, formal analysis, software. **Misaki Hasegawa**: writing – review and editing, data curation, investigation. **Willy Shun Kai Bong**: investigation, writing – review and editing. **Koji Hiraoka**: investigation, formal analysis, writing – review and editing. **Kazuo Yamamoto**: supervision, writing – review and editing. **Yutaka Ito**: project administration, writing – review and editing. **Yoshiya Fujiwara**: project administration, funding acquisition, supervision, writing – review and editing. **Yoshitaka Tateyama**: funding acquisition, writing – review and editing, supervision, methodology, project administration. **Takuhiro Miyuki**: project administration, funding acquisition, supervision, writing – review and editing.

## Funding

This work was financially supported by New Energy and Industrial Technology Development Organization (JPNP23005), Japan Society for the Promotion of Science (23K13837, 23H00241, 25K00078), Acquisition, Technology & Logistics Agency (JPJ004596), Iketani Science and Technology Foundation, Daiko Foundation.

## Ethics Approval Statement

Ethics approval was not required for this study.

## Conflicts of Interest

The authors declare no conflicts of interest.

## Supporting information




**Supporting File 1**: advs77717‐sup‐0001‐SuppMat.pdf.

**Supporting File 2**: advs77717‐sup‐0002‐SuppMat.docx.

## Data Availability

The data that support the findings of this study are available from the corresponding author upon reasonable request.
