# Supplementary material for: Visualizing a Li‐Depleted Amorphous Cathode–Electrolyte Interphase in Sulfide Solid‐State Batteries Using In Situ Cryogenic Electron Microscopy
Source: Adv Sci (Weinh). 2026 Sep 13:e77717. Online ahead of print. doi: 10.1002/advs.77717 (PMC13572859; doi:10.1002/advs.77717)
Supplement: Supplementary file 1 [file ADVS-9999-e77717-s001.pdf]

Supporting Information

Visualizing a Li-depleted amorphous cathode–electrolyte interphase in sulfide solid-state batteries using in situ cryogenic electron microscopy

Yuki Nomura,[1,] Ryoma Sasaki,[2] Huu Duc Luong,[2] Misaki Hasegawa,[1] Willy Shun Kai Bong,[3] Koji Hiraoka,[1] Kazuo Yamamoto,[1] Yutaka Ito,[3] Yoshiya Fujiwara,[3] Yoshitaka Tateyama,[2] Takuhiro Miyuki[3]*

[1]Nanostructures Research Laboratory, Japan Fine Ceramics Center, 2–4–1 Mutsuno, Atsuta-ku, Nagoya, Aichi 456–8587, Japan.

[2]Laboratory for Chemistry and Life Science, Institute of Integrated Research, Institute of Science Tokyo, R1-25, 4259 Nagatsuta-cho, Midori-ku, Yokohama 226-8501, Japan.

[~~2~~3]Consortium for Lithium Ion Battery Technology and Evaluation Research Center, 1–8–31 Midorigaoka, Ikeda, Osaka 563–8577, Japan.

* To whom correspondence should be addressed. E-mail: y_nomura@jfcc.or.jp

Table of Contents

Supporting Note 1. Experimental methods.

Supporting Note 2. Absence of significant changes in the O distribution.

Supporting Note 3. Electronic state of S.

Fig. S1. Compensating currents applied during FIB milling and plasma cleaning.

Fig. S2. EIS analysis of a standard solid-state electrochemical cell under constant-voltage holding at 3.7 V vs. Li–In.

Fig. S3. Low-magnification ADF-STEM images before and after charging to 3.5 and 3.7 V vs. Li–In.

Fig. S4. ADF-STEM images of the NCM523/LPSC interface before and after charging.

Fig. S5. Comparison of the EEL spectra of LPSC and NCM523 around the Li–K edge.

Fig. S6. Comparison of nanobeam electron diffraction (NBD) patterns obtained from LPSC.

Fig. S7. Low-magnification ADF-STEM images of the $LiNbO_y$-coated NCM523/~~$LiNbO_y$/~~LPSC interface before and after charging at 3.7 V vs. Li–In.

Fig. S8. In situ imaging of ~~CEI formation between~~Li depletion at the $LiNbO_y$-coated NCM523~~ and ~~/LPSC interface.

Fig. S9. Effect of $LiNbO_y$ coating on interfacial degradation as evaluated via EIS using a standard solid-state electrochemical cell.

Fig. S10. Crystallinity analysis ~~of~~ at the ~~CEI between~~ $LiNbO_y$-coated NCM523~~ and~~ /LPSC before charging.

Fig. S11. *LC* analysis at the ~~CEI between~~ $LiNbO_y$-coated NCM523~~ and ~~/LPSC interface.

Fig. S12. Validation of the NNP-PFP potential against DFT calculations for Li-deficient Li_xPS_5Cl structures.

Fig. S13. Convex hull obtained by DFT simulation.

Fig. S14. Mean squared displacement (MSD) of Li ions in Li_xPS_5Cl at various temperatures obtained from MD simulations.

Supporting Note 1. Experimental methods.

Materials. Argyrodite-type LPSC (MSE Supplies LLC), which exhibits high ionic conductivity (>10^{-3} S cm^{-1} at 25 °C), was employed as the SE. Polycrystalline NCM523 (D50 = 5.2 µm) was used as the cathode active material. To prepare the $LiNbO_y$ coating layer, a Li–Nb double alkoxide was dissolved in anhydrous ethanol and used as the precursor. After adding bare NCM523 particles to the precursor solution, the mixture was processed using a high-pressure gas-flow fluidized coating machine (JD-1, Kawata) to fabricate $LiNbO_y$ coatings with an average thickness of ~9 nm. The coating thickness was controlled by adjusting the number of coating cycles.~~, as described in the literature.[1]~~ All samples were subsequently ~~heat~~ heat-treated in an oxygen atmosphere at 300 °C for 3 h to obtain amorphous $LiNbO_y$ coating layers. The morphology, local thickness distribution, and surface coverage of $LiNbO_y$ coatings prepared using the same procedure were systematically characterized by cross-sectional TEM and SEM-AES in a previous study.[1]

Solid-state battery cell assembly using standard electrochemical cells. The composite cathode was prepared by mechanically blending bare or $LiNbO_y$-coated NCM523 with LPSC at a mass ratio of 80:20 using an agate mortar and pestle until a homogeneous mixture was obtained. The resulting mixture was assembled into SSB cells with the configuration [NCM523–LPSC/LPSC/Li–In]. LPSC (100 mg) and composite powder (4 mg) were loaded into a die with a diameter of 10 mm and compacted under a uniaxial pressure of 370 MPa. On the counter-electrode side, a 150-µm-thick In–Li–In trilayer was placed such that the Li–In alloy was in contact with the LPSC surface. The resulting three-layer pellet was further consolidated by pressing at 90 MPa and subsequently sandwiched between stainless-steel pins serving as current collectors. All procedures were performed inside an Ar-filled glovebox with a dew point below −70 °C. Electrochemical cycling and EIS measurements (Figure S2 and S9) were performed under an applied stack pressure of 100 MPa.

Millimeter-scale ~~S~~solid-state battery cell assembly for in situ transmission electron microscopy. Similar materials as those used in the standard electrochemical cells were employed for the cathode, SE, and counter electrode. To fabricate the separator layer, 50 mg of LPSC powder was uniaxially pressed in a cylindrical mold to form a pellet with a diameter of 10 mm. Bare or $LiNbO_y$-coated NCM523 powder was then gently distributed onto one surface of the pellet. The stacked components were densified by uniaxial hot pressing at 120 °C under a pressure of 370 MPa. A 150-µm-thick In–Li–In trilayer was subsequently placed onto the other flat end ~~opposite side~~ of the pellet and further consolidated by pressing at 90 MPa. The resulting NCM523/LPSC/Li–In assembly was removed from the die and transferred to a sputtering system, where a Pt current collector was deposited on the NCM side without air exposure. The fabricated pellet was then sectioned into pieces measuring ~1 mm × 1 mm × t0.5 mm using a razor blade, and Cu electrodes were attached to both faces using a solvent-free silver paste. The geometry of the fully assembled cell is shown in Figure 2b and c.

Scanning transmission electron microscopy with electron energy-loss spectroscopy. Lamellae for STEM analysis were prepared using a FIB system (FB-2100, Hitachi High-Technologies Corp.) that was operated at an accelerating voltage of 40 kV, thinning the cathode region to a final thickness of ~150 nm. The STEM-EELS measurements were performed using a 300-kV transmission electron microscope (JEM-ARM300F2 GRAND ARM2, JEOL Ltd.) equipped with an EEL spectrometer (Gatan Imaging Filter Continuum-K3, Gatan Inc.). The specimens were mounted on a vacuum-transfer TEM holder incorporating dual biasing electrodes and a cryogenic and heating stage (Elecryo holder, Mel-Build Corp.). The holder geometry is illustrated in Figure 2f. All transfer processes were conducted either under an Ar atmosphere or under vacuum conditions to avoid air exposure. EEL spectra were acquired with an energy dispersion of 0.35 eV pix^{-1} using a probe current of ~3.1 pA. Spectrum imaging was performed with a dwell time of 3.4×10^{-4} s pix^{-1} and spatial sampling interval of 6 nm. A single-electron-counting direct-detection complementary metal–oxide–semiconductor (CMOS) detector (K3 camera, Gatan Inc.) was used for spectrum acquisition. Each STEM-EELS dataset was collected using the multipass in situ spectrum imaging mode implemented in GMS 3.6, where 55 sequential scans were accumulated. The electron dose for a STEM-EELS acquisition was ~100 e^{-} $Å^{-2}$.

Energy-filtered scanning nanobeam electron diffraction. EF-SNBD was performed using the same transmission electron microscope and specimen holder as those used for STEM-EELS. The probe current and spatial sampling interval were 1.2 pA and 6 nm pix^{-1}, respectively. The dwell time was set to 1.2×10^{-2} s pix^{-1} when obtaining the results shown in Figure 4b, 6b, 6c, S10b, and S10c and to 3.4×10^{-3} s pix^{-1} when obtaining the results shown in Figure 4c. An energy slit width of 20 eV centered on the zero-loss peak was used for energy filtering. The same single-electron-counting direct-detection CMOS detector (K3 camera, Gatan Inc.) was used to acquire the NBD patterns. The electron doses for the (EF-)SNBD acquisitions were ~25 or 7 e^{-} $Å^{-2}$.

Quantification of Li concentration and evaluation of uncertainty. The Li concentration in LPSC was evaluated from the integrated intensity of the Li–K edge. A power-law function was used for background subtraction. The background was estimated using the energy ranges of 54.95–58.10 eV and 74.90–78.05 eV, and the Li–K signal was integrated over the range of 58.10–64.40 eV after background subtraction. The same background-subtraction procedure was applied to all spectra used for the quantitative analysis. The resulting Li–K intensity was normalized to that measured in the bulk LPSC region of the same dataset, which was assigned to the stoichiometric composition of Li_6PS_5Cl. The specimen thickness within the analyzed LPSC region was approximately 0.6 λ (λ: the inelastic mean free path of 300 kV electrons), with a variation of less than 2%; therefore, the influence of local thickness variations on the relative Li–K intensity was minor. In addition, no other core-loss edges in LPSC overlap with the Li–K edge. Because the specimen thickness was nearly constant, any residual influence of plural scattering is expected to affect the spectra uniformly and therefore have only a minor effect on the relative Li-concentration profile. As shown in Figure 1, electron irradiation can cause a Li loss of up to 10% near the practical upper dose limit of 400 e^{-} $Å^{-2}$. For quantitative comparison of the Li-concentration profiles (Figure 3g, 5f, and 5g),

the Li–K intensity was normalized to that of the bulk LPSC region within the same dataset to minimize the influence of beam-induced Li loss and probe-current decay. Thus, the quantified Li concentration represents the local Li content relative to the bulk LPSC subjected to the same electron-irradiation conditions. As an empirical measure of the precision of the Li-concentration analysis, the mean ± standard deviation values in the bulk LPSC regions were 6.0 ± 0.17, 6.0 ± 0.28, and 6.0 ± 0.21 for Figures 3g, 5f, and 5g, respectively, corresponding to relative standard deviations of approximately 2.8–4.3%.

Electrochemical measurements. Charge–discharge tests using a standard electrochemical cell were conducted at 25 and 80 °C with a battery testing system (HJ1020mSD8, Meiden Hokuto) under a constant current density of 9 mA g^{-1}, with cutoff voltages of 3.1, 3.4, and 3.7 V. Charge–discharge measurements inside the transmission electron microscope were performed using a potentio/galvanostat (SP-200, Bio-Logic Science Instruments) at a constant current of 200 or 400 nA, followed by a constant voltage hold at 3.5 and 3.7 V for 1 h at 80 °C. EIS was performed using the same potentio/galvanostat with an applied voltage amplitude of 10 mV over a frequency range ~~from~~ of 3 MHz to 100 mHz.

Machine-learning-based molecular dynamics simulations. A 2 × 2 × 2 supercell of crystalline Li_6PS_5Cl (32 formula units) with a S^{2-}/Cl^- anion disorder of 50% based on the argyrodite structure was constructed as the reference bulk model.[2] Li-deficient structures were generated by randomly removing Li atoms from the supercell to obtain Li_4PS_5Cl (64 Li atoms removed per supercell) and Li_2PS_5Cl (128 Li atoms removed per supercell). All structural relaxations and MD simulations were carried out using a universal machine-learning interatomic potential (NNP-PFP version 8.0.0),[3] which is commercially available in Matlantis with Grimme's D3 dispersion correction.[4] The PFP was trained on more than 10^7 calculations including bulk, cluster, surface, and disordered structures of both organic and inorganic systems using the Perdew–Burke–Ernzerhof (PBE) level of theory.[5] The simulation cells were maintained overall charge-neutral when Li atoms were removed. Therefore, removal of a neutral Li atom corresponds to simultaneous removal of Li^+ and the associated electron. The machine-learning interatomic potential does not explicitly describe electronic degrees of freedom; instead, changes in the effective interatomic interactions associated with electronic redistribution upon Li removal are implicitly incorporated through the potential trained on DFT energies and forces. The results of the NNP-PFP calculations were compared with DFT calculations to verify the NNP accuracy. The DFT calculations were implemented in the Vienna *Ab initio* simulation package (VASP)[6,7] using the projector-augmented-wave (PAW) method and PBE generalized gradient approximation[5] combined with Grimme D3 correction. A unit cell of $Li_{24}P_4S_{20}Cl_4$ was chosen as the parent structure for this examination. A kinetic cutoff energy of 520 eV and a *k*-point mesh of 2 × 2 × 2 were set for all calculations. The energies and atomic forces of 500 structures—extracted from the trajectories of the Langevin thermostat simulation in the isothermal–isobaric (*NpT*) ensemble at 500, 600, 700, 800, and 900 K[4–7] with different Li-concentration Li_xPS_5Cl (x = 6, 4, 2)—were compared with those predicted by the NNP-PFP. Geometry and lattice parameters were optimized until the maximum residual force on each atom was less than 0.03 eV $Å^{-1}$. MD simulations were performed with a time step of 1 fs. Each system was first

equilibrated in the *NpT* ensemble at 1 atm using an isotropic Parrinello–Rahman barostat for 200 ps,[8] followed by production runs in the canonical (*NVT*) ensemble using a Nosé–Hoover thermostat for 100–500 ps.[9,10] Li self-diffusion coefficients were obtained from the MSDs of Li atoms using the Einstein relation in the linear diffusive regime. The MSD calculation and linear fitting were performed using KINISI software, which uses Bayesian linear regression.[11,12] Activation energies were extracted from Arrhenius fits of the temperature-dependent diffusion coefficients. Simulated powder diffraction patterns were generated from representative configurations sampled from equilibrated MD trajectories to assess the evolution of crystallinity. Li–Li connectivity networks were constructed by connecting Li atoms separated by less than 3.5 Å, a cutoff chosen to identify the nearest-neighbor Li connectivity in the argyrodite framework~~, in order to evaluate changes in the diffusion pathway connectivity with decreasing Li content~~. This analysis was used only as a qualitative visualization of changes in the Li sublattice with decreasing Li content; Li transport was evaluated quantitatively from the MD-derived self-diffusion coefficients.

Supporting Note 2. Absence of significant changes in the O distribution.

The distributions of O ~~in Figure 3d–f~~ showed no significant changes, as illustrated in Figure 3d–f. Possible explanations include (1) differences in the electrochemical cycling conditions, (2) the amount of O_2 gas released, (3) differences in the cell configuration, and (4) the low signal-to-noise ratio (SNR) of the O distribution. Regarding (1), previous ToF-SIMS studies analyzed electrodes that were cycled 100 times or charged to 5.0–6.0 V vs. Li–In.[13,14] In contrast, the present study focused on the first charged state at 3.7 V vs. Li–In, where the interfacial conditions were different. Moreover, regarding (2), the amount of O_2 gas released is expected to be limited. Compared with Ni-rich NCM (e.g., NCM811), the contraction of the (003) planes of NCM523 at 4.3 V vs. Li (3.7 V vs. Li–In) is relatively modest, suggesting that extensive O_2 gas release associated with the H2–H3 phase transition is unlikely under the present charging conditions.[15] Indeed, O_2 gas release from NCM523 in SSBs has been detected only above 4.55 V vs. Li at first charge,[16] suggesting that the amount of ~~released~~ O_2 released was not significant. In contrast to the above-discussed electrochemical factors, (3) differences in cell configuration may also have played a role. In a sealed standard electrochemical cell, spherical NCM523 particles are three-dimensionally covered by the SE, and the O_2 gas released from the NCM523 particles is directed toward the SE through their surfaces, where it interacts with the SE. In contrast, in our cell, charging was performed in a thinned lamella configuration; therefore, most of the O_2 gas was likely ~~to have~~ released directly into the TEM column rather than interacting with the SE. Furthermore, when considering (4) under the low-dose~~, damage-free imaging~~ conditions used to minimize electron-beam-induced damage in this study, the O distribution exhibited a poor SNR. This limitation may have masked subtle variations, resulting in the absence of an apparent difference. Although no significant change in the O distribution was noted under the present low-dose conditions, oxygen-mediated reactions between highly charged NCM523 and LPSC cannot be completely excluded and may contribute to the formation of oxygen-containing interfacial products and the remaining interfacial resistance.

Supporting Note 3. Electronic state of S.

As suggested by previous XPS studies,[17] during the oxidative decomposition of LPSC, electrons are expected to be removed from the S 3p orbitals, oxidizing S^{2-} species in the PS_4 units to polysulfide-like or S^0 states (e.g., P–S–S–P– and~~,~~ –S–S– motifs~~, etc.~~). Ideally, such oxidation can be verified by analyzing the S–K edge; however, because the S–K edge lies around 2,470 eV, its analysis by EELS requires a significant electron dose. Therefore, the S–K edge could not be analyzed under the low-dose ~~and damage-free~~ conditions employed in this study. As an alternative indicator, we examined the integrated intensity of the S–L edge; however, no significant change was detected (third column of panels from the right in Figure 3d–f). Because changes in the S chemical state do not necessarily produce a substantial change in the integrated S–L intensity, the absence of a detectable intensity change does not exclude the formation of polysulfide-like or S^0 species.~~This result is consistent with the fact that polysulfide-like and S^0 species remain solid below room temperature, and thus their formation may not produce a detectable change in the integrated intensity of the S–L edge.~~

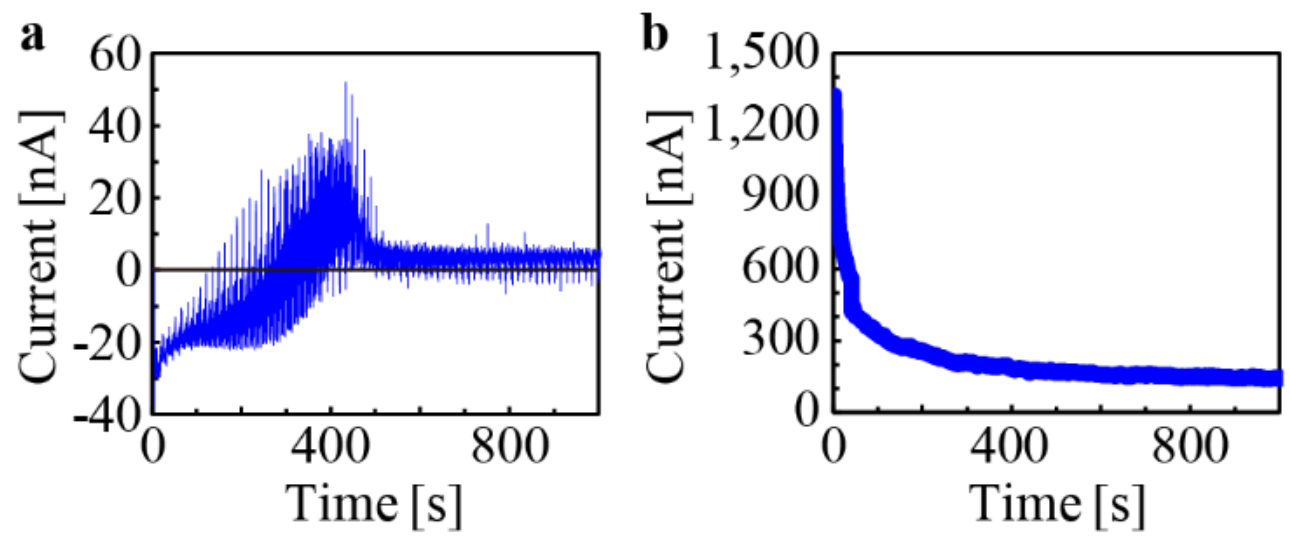

Figure S1. Compensating currents applied during (a) FIB milling and (b) plasma cleaning.

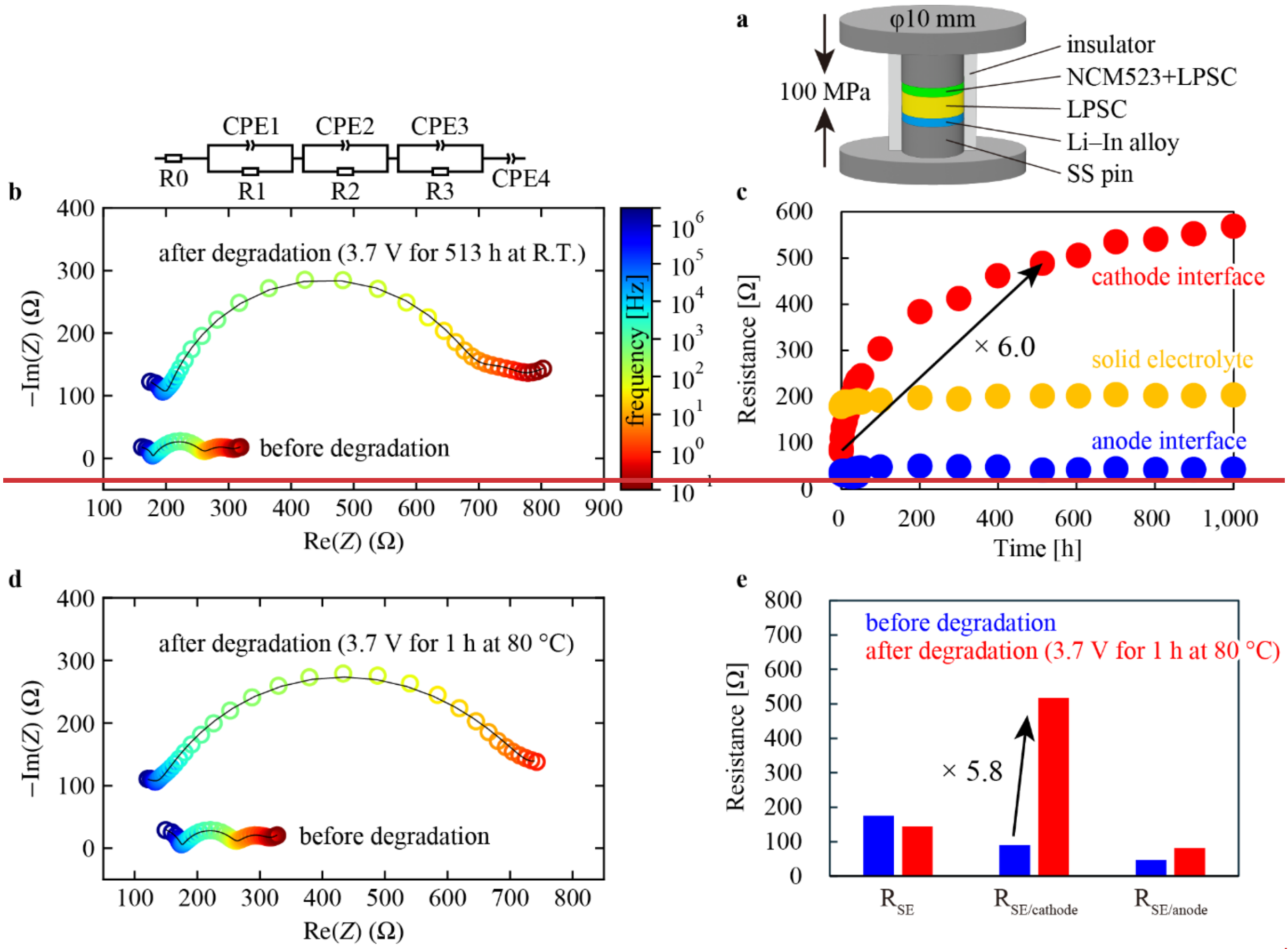
a
φ10 mm
100 MPa
insulator
NCM523+LPSC
LPSC
Li–In alloy
SS pin
CPE1
CPE2
CPE3
R0
R1
R2
R3
CPE4
b
after degradation (3.7 V for 513 h at R.T.)
before degradation
−Im(Z) (Ω)
Re(Z) (Ω)
frequency [Hz]
c
cathode interface
× 6.0
solid electrolyte
anode interface
Resistance [Ω]
Time [h]
d
after degradation (3.7 V for 1 h at 80 °C)
before degradation
−Im(Z) (Ω)
Re(Z) (Ω)
e
before degradation
after degradation (3.7 V for 1 h at 80 °C)
× 5.8
Resistance [Ω]
R_SE
R_SE/cathode
R_SE/anode

226

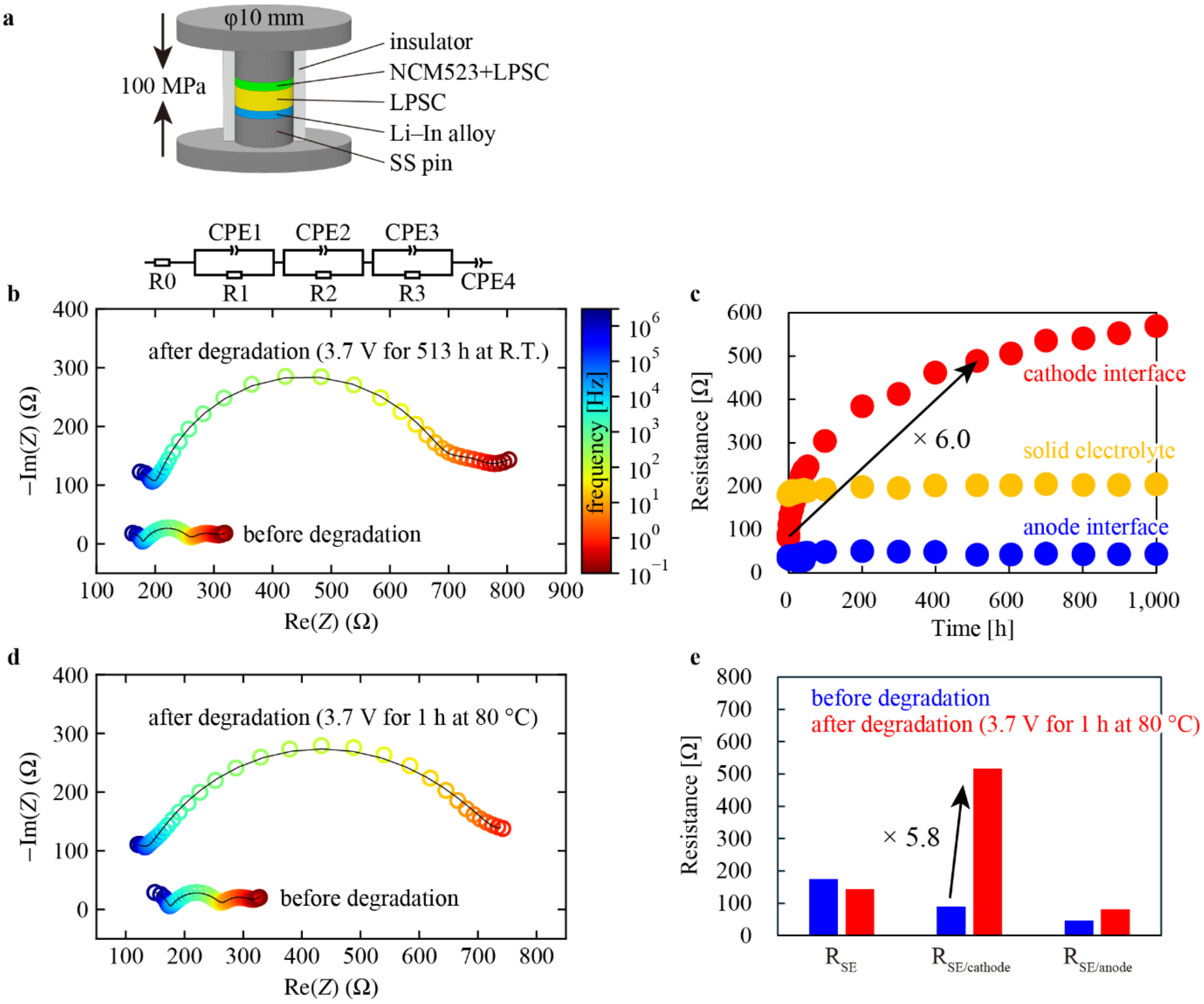

Figure S2. EIS analysis of a standard solid-state electrochemical cell under constant-voltage holding at 3.7 V vs. Li–In. (a) Schematic of a standard solid-state electrochemical cell. (b) Comparison of Nyquist plots obtained at room temperature before degradation (0 h) and after prolonged holding at 3.7 V vs. Li–In for 513 h. Colored symbols represent the experimental EIS data, and the solid black lines correspond to the fitted curves obtained using the equivalent circuit shown above the panel. (c) Time evolution of the three resistances associated with the SE, cathode interface, and anode interface during holding at 3.7 V vs. Li–In at room temperature. (d) Comparison of Nyquist plots obtained at room temperature before degradation (0 h) and after holding at 3.7 V vs. Li–In for 1 h at 80 °C. (e) Comparison of the three resistance components extracted from the EIS spectra before and after degradation.

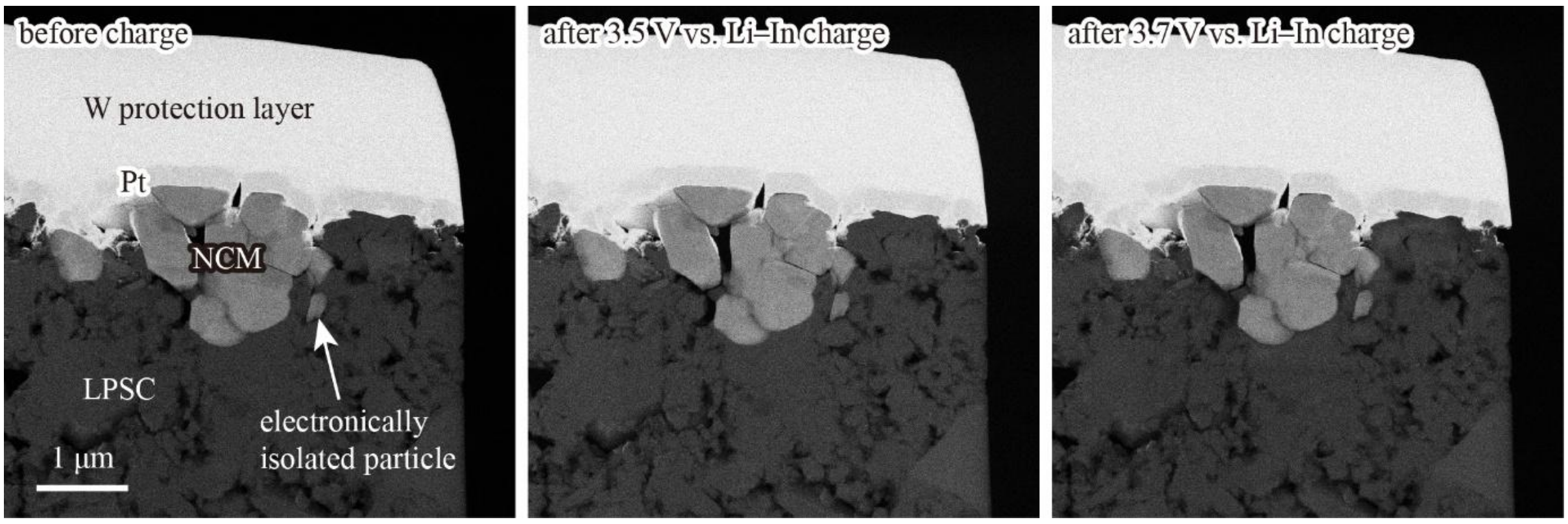

Figure S3. Low-magnification ADF-STEM images before and after charging to 3.5 and 3.7 V vs. Li–In.

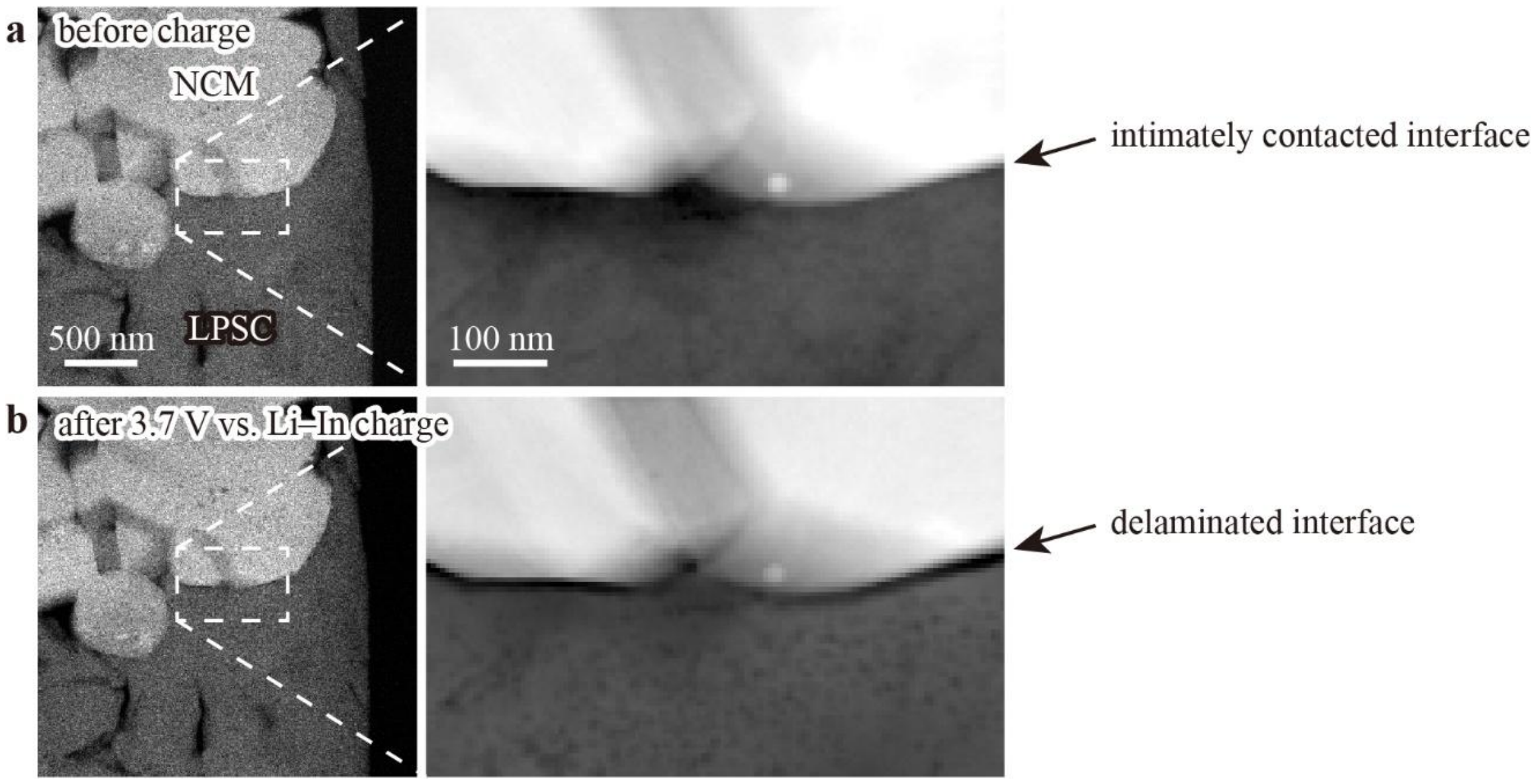

Figure S4. ADF-STEM images of the NCM523/LPSC interface before and after charging. (a) Before charging, the interface was intimately contacted. (b) After charging to 3.7 V vs. Li–In, the interface became delaminated. Magnified views are shown on the right.

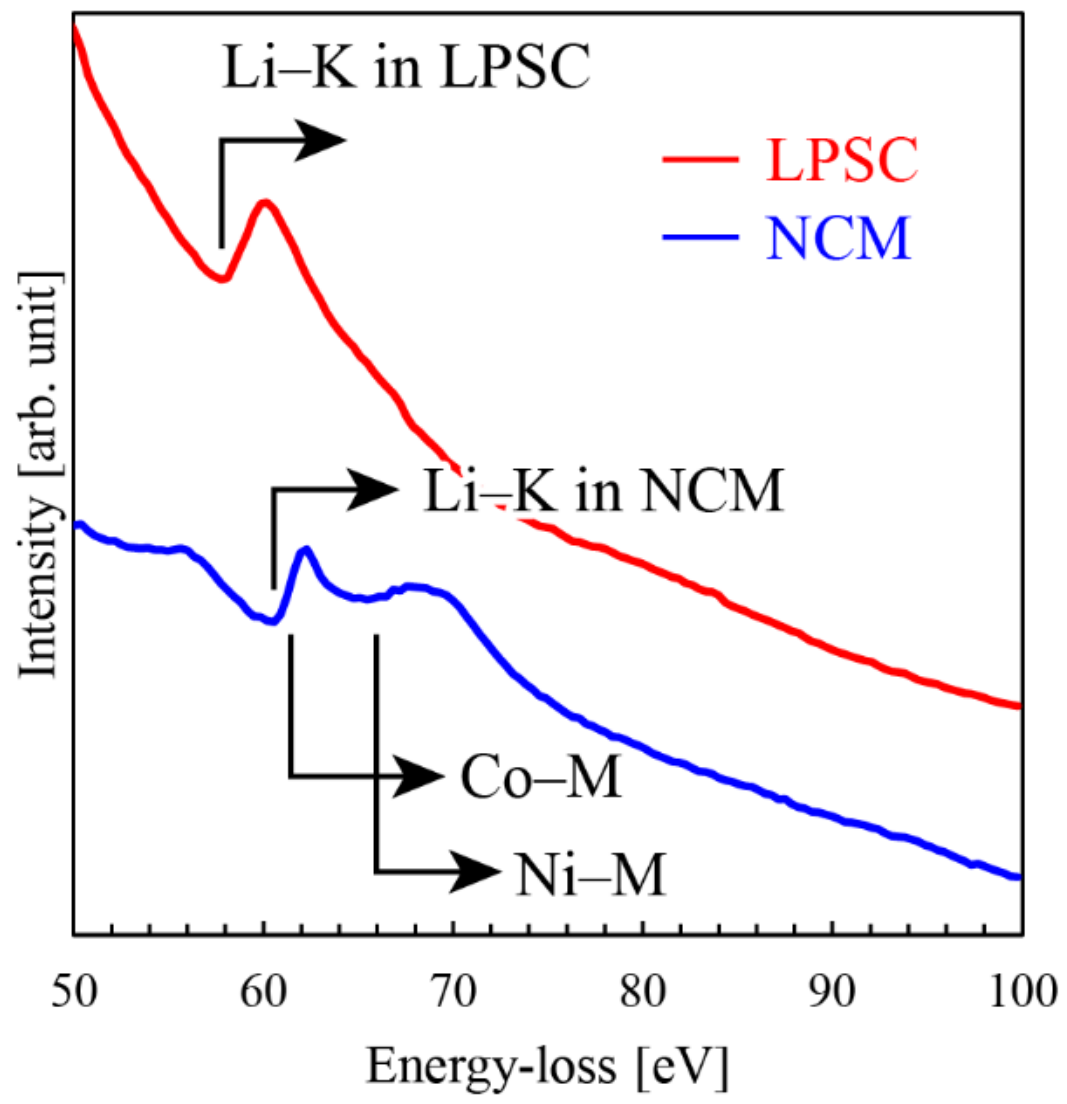

Figure S5. Comparison of the EEL spectra of LPSC and NCM523 around the Li–K edge. The Li–K edge appears at ~58 eV in LPSC and at ~60 eV in NCM. Moreover, the NCM523 spectrum includes the Co–M and Ni–M edges, as illustrated in the figure.

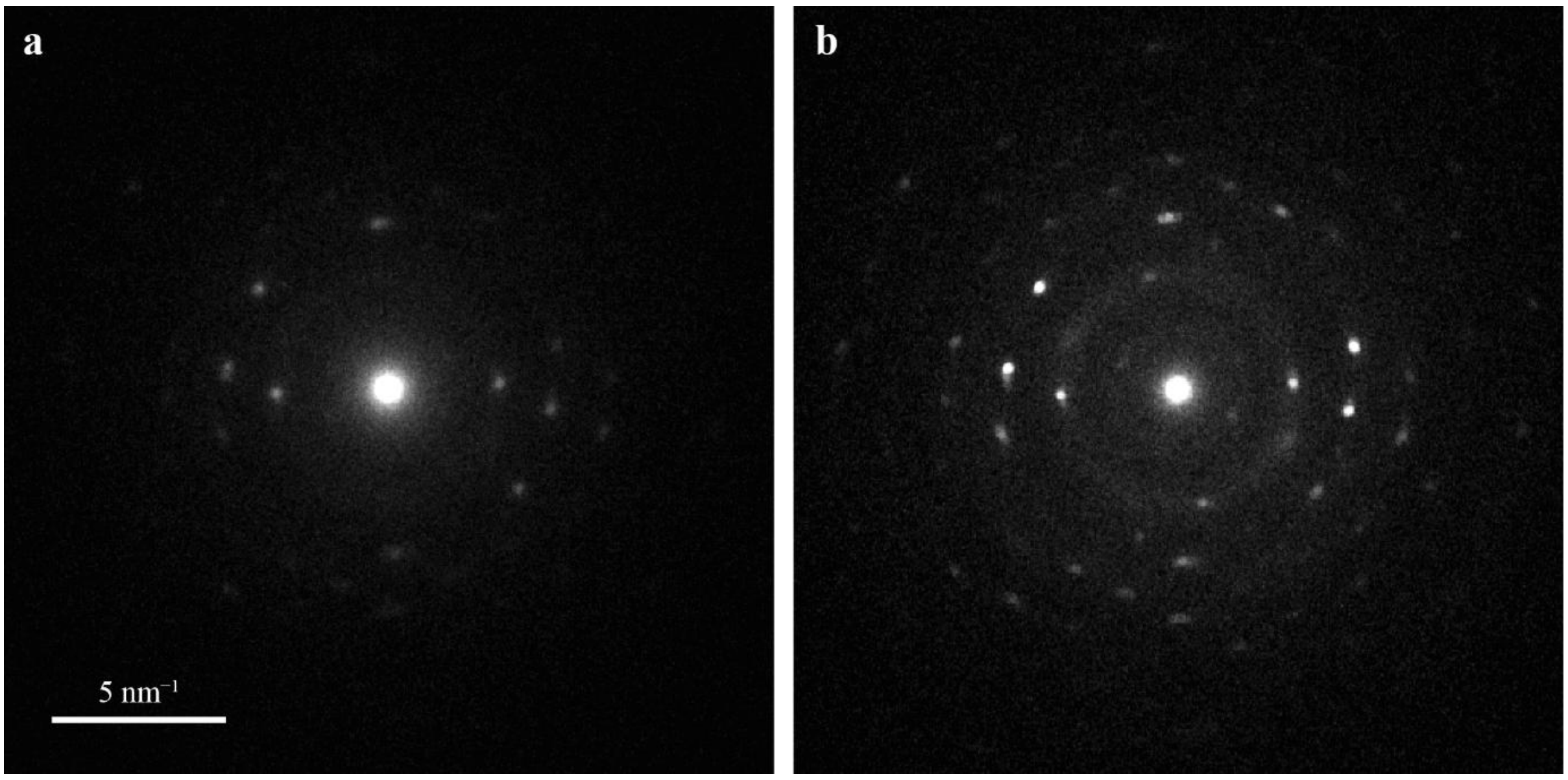

Figure S6. Comparison of NBD patterns obtained from LPSC. (a) NBD pattern acquired without an energy filter. (b) NBD pattern acquired with an energy filter (E_{loss} = 0 ± 10 eV).

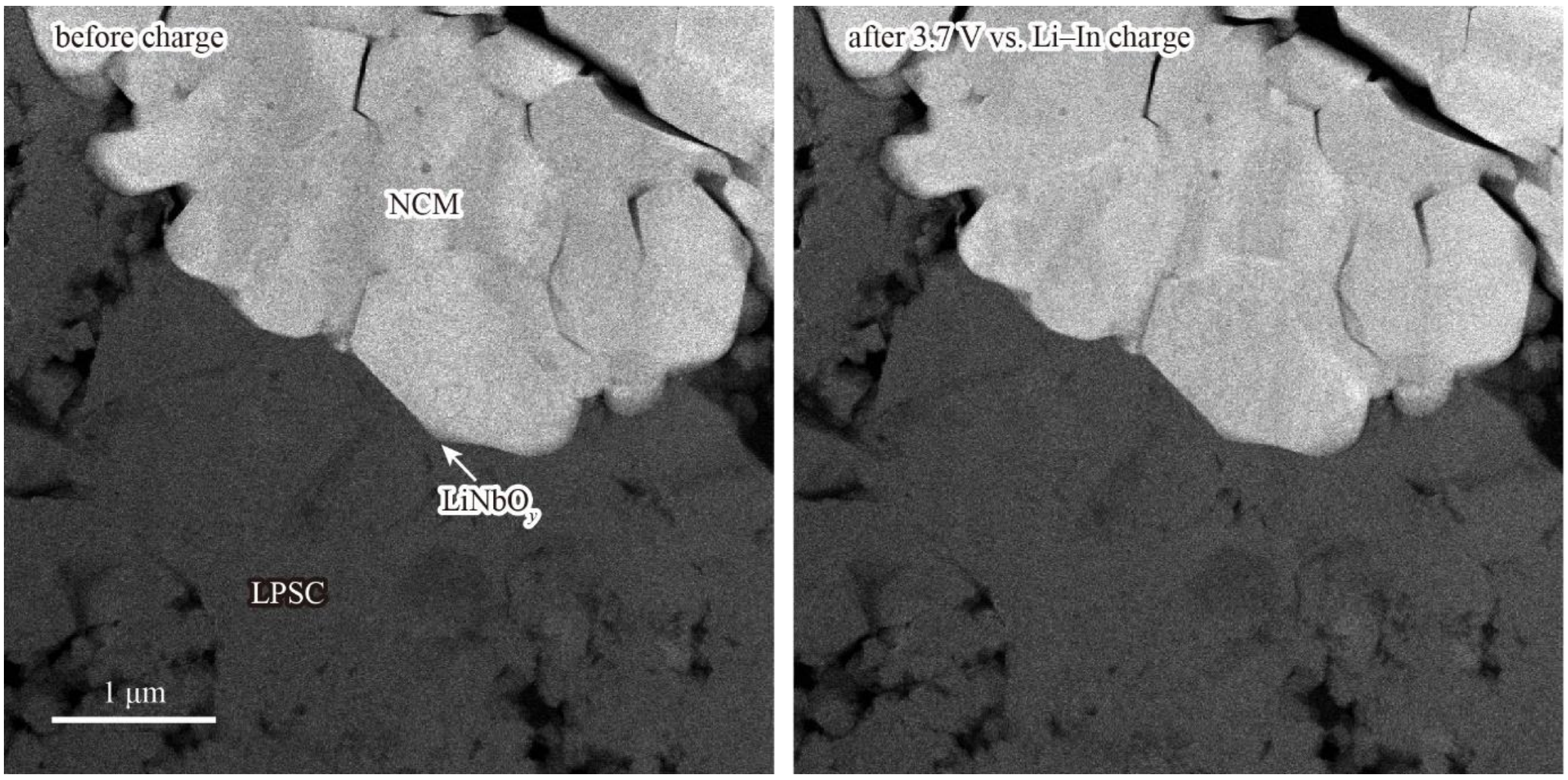

Figure S7. Low-magnification ADF-STEM images of the LiNbO$_y$-coated NCM523/ LPSC~~NCM523/LiNbO$_y$/LPSC~~ interface before and after charging at 3.7 V vs. Li–In.

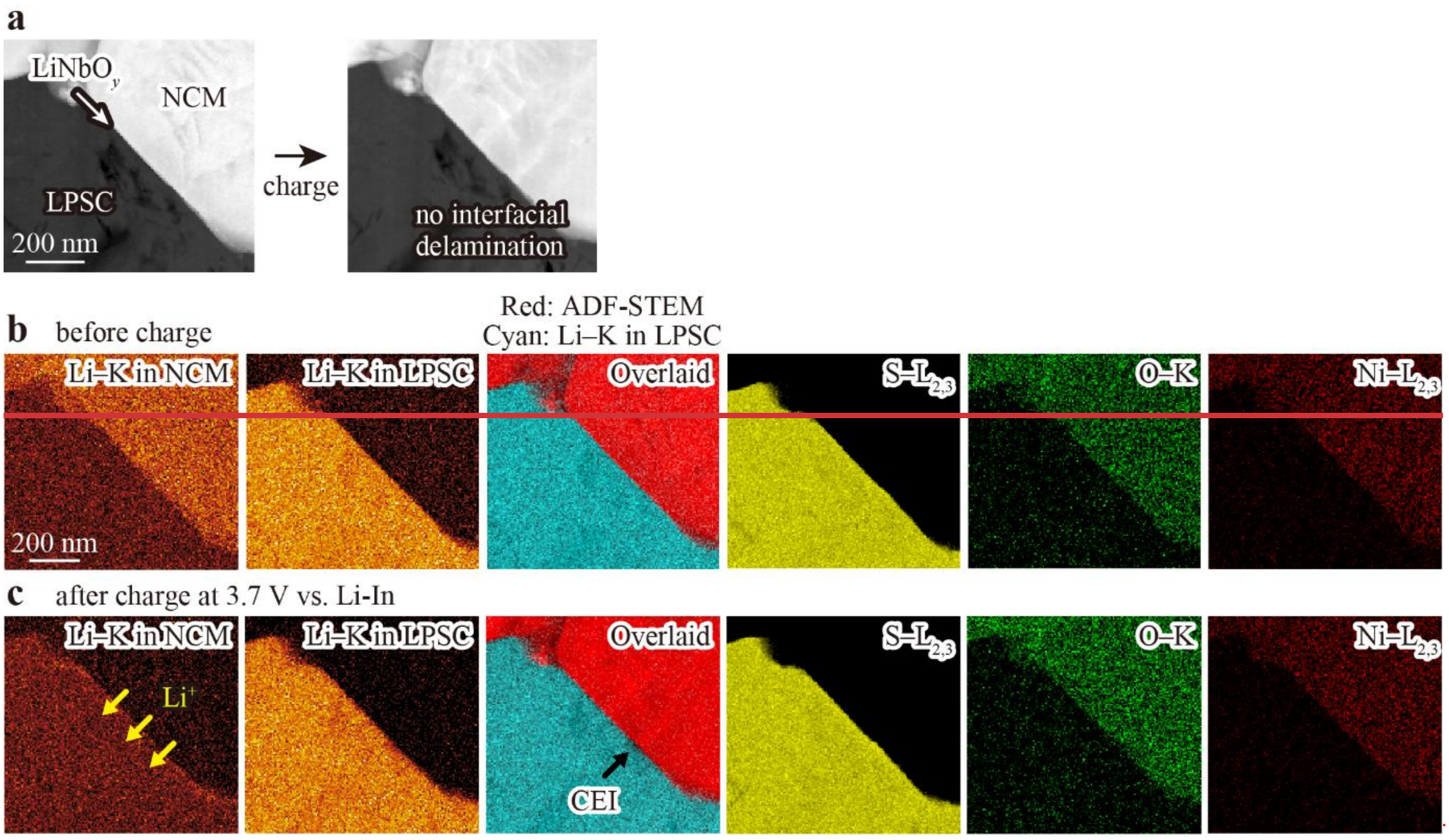

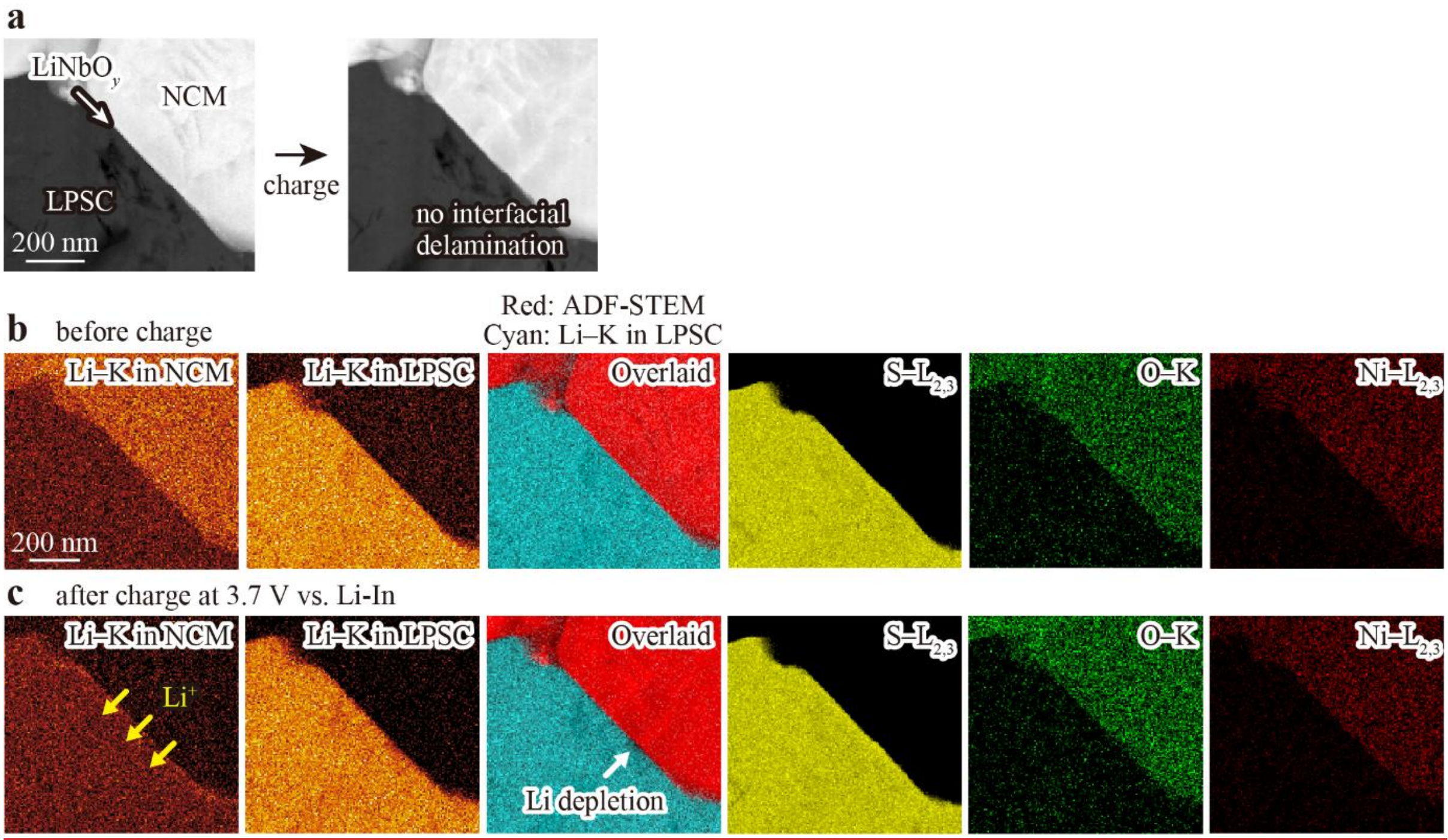

Figure S8. In situ imaging of Li depletion at the~~CEI formation between~~ LiNbO$_y$-coated NCM523~~ and ~~/LPSC interface. (a) ADF-STEM images before and after charging. (b), (c) Elemental distributions (b) before and (c) after charging at 3.7 V vs. Li–In.

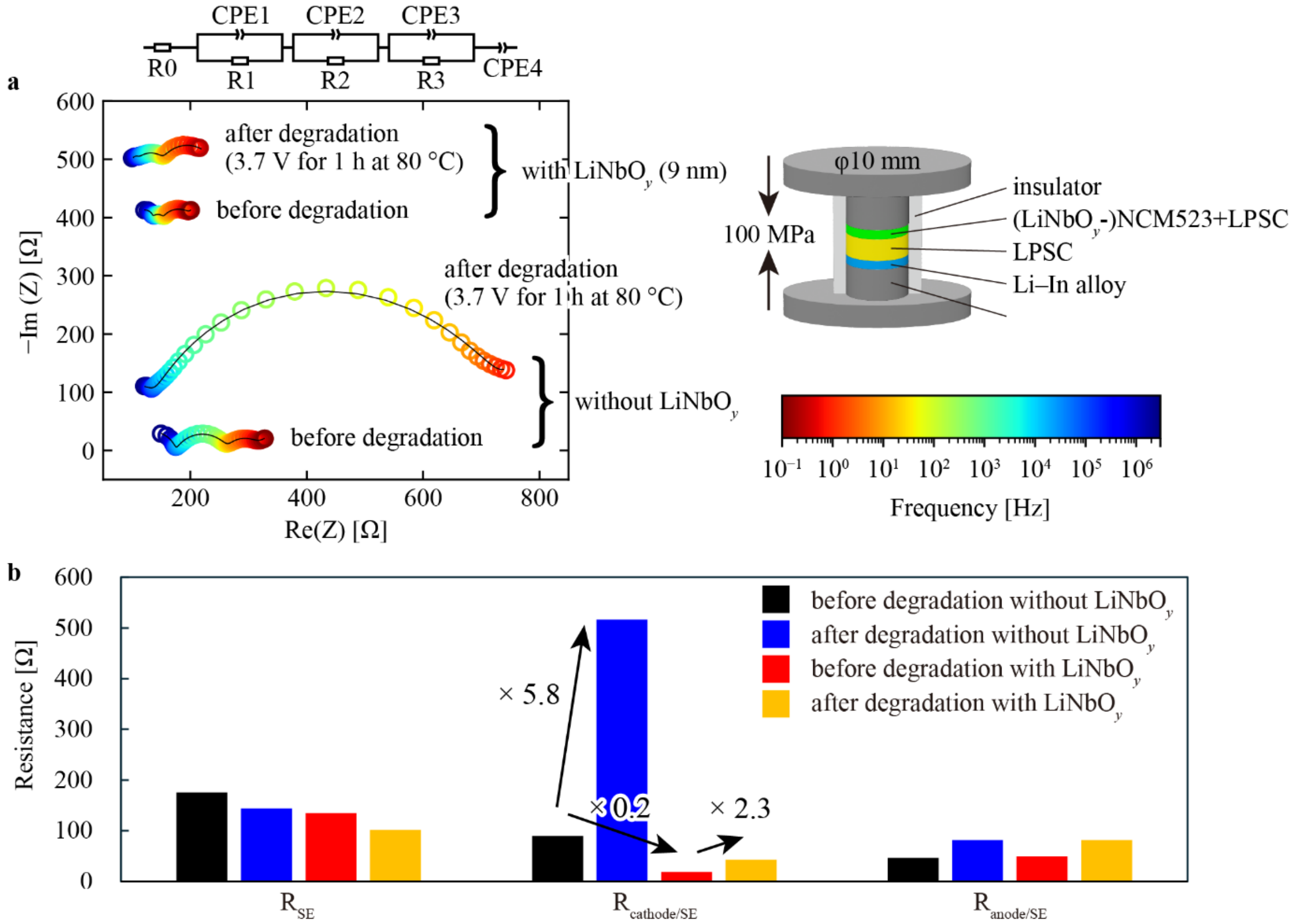

Figure S9. Effect of $LiNbO_y$ coating on interfacial degradation as evaluated via EIS using a standard solid-state electrochemical cell. (a) Nyquist plots of solid-state cells with and without a $LiNbO_y$ coating (~9 nm) on the NCM cathode, which were measured at room temperature before degradation and after holding at 3.7 V vs. Li–In for 1 h at 80 °C. Colored symbols represent the experimental EIS data, and the solid black lines correspond to the fitted curves obtained using the equivalent circuit shown above the panel. (b) Resistance components extracted from the EIS fitting, including the SE resistance (R_{SE}), cathode interfacial resistance ($R_{cathode/SE}$), and anode interfacial resistance ($R_{anode/SE}$), before and after degradation for $LiNbO_y$-coated and uncoated samples.

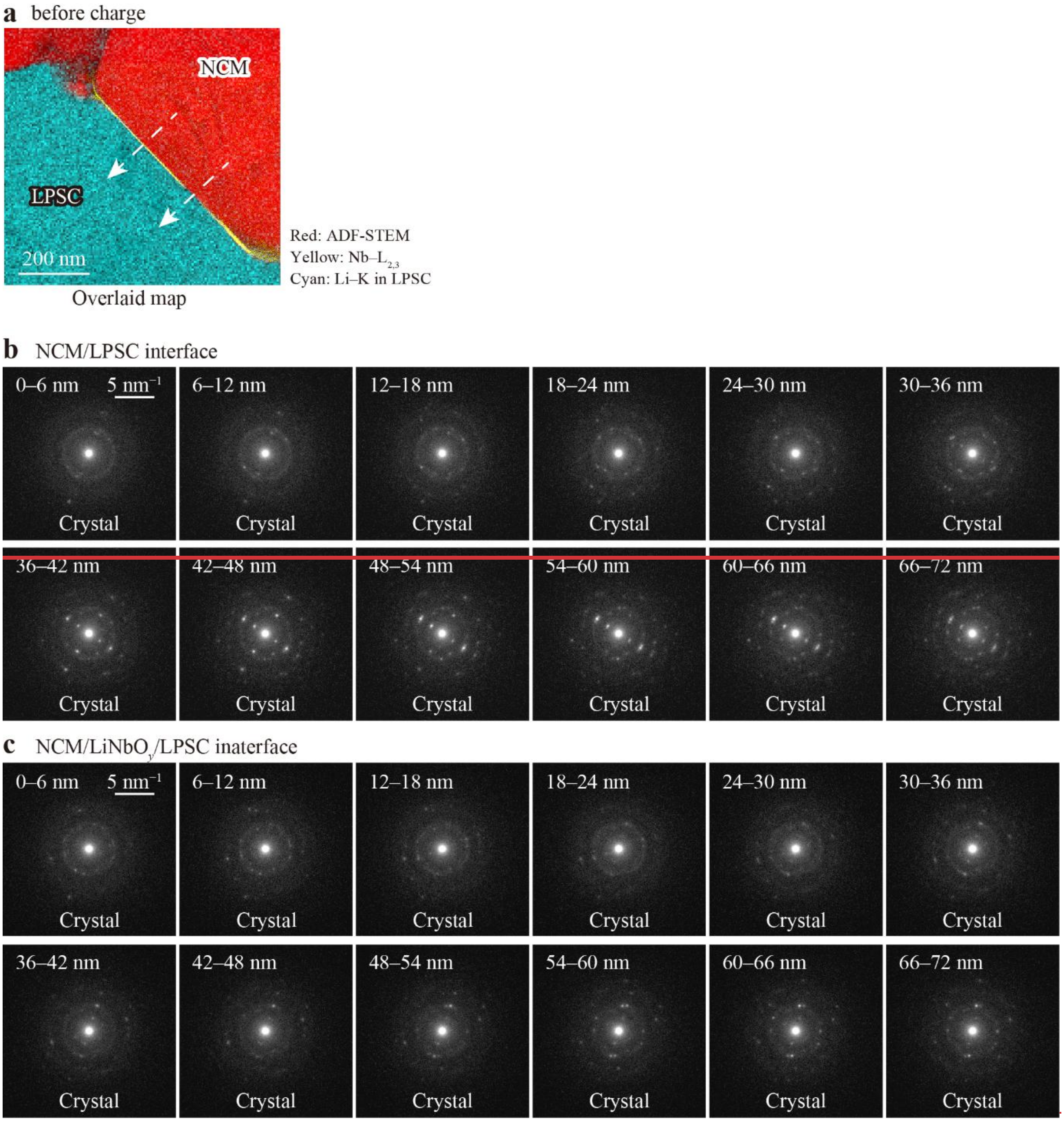
a before charge
NCM
LPSC
200 nm
Overlaid map
Red: ADF-STEM
Yellow: Nb–L2,3
Cyan: Li–K in LPSC
b NCM/LPSC interface
0–6 nm
5 nm−1
6–12 nm
12–18 nm
18–24 nm
24–30 nm
30–36 nm
36–42 nm
42–48 nm
48–54 nm
54–60 nm
60–66 nm
66–72 nm
Crystal
c NCM/LiNbOy/LPSC inaterface
0–6 nm
5 nm−1
6–12 nm
12–18 nm
18–24 nm
24–30 nm
30–36 nm
36–42 nm
42–48 nm
48–54 nm
54–60 nm
60–66 nm
66–72 nm
Crystal

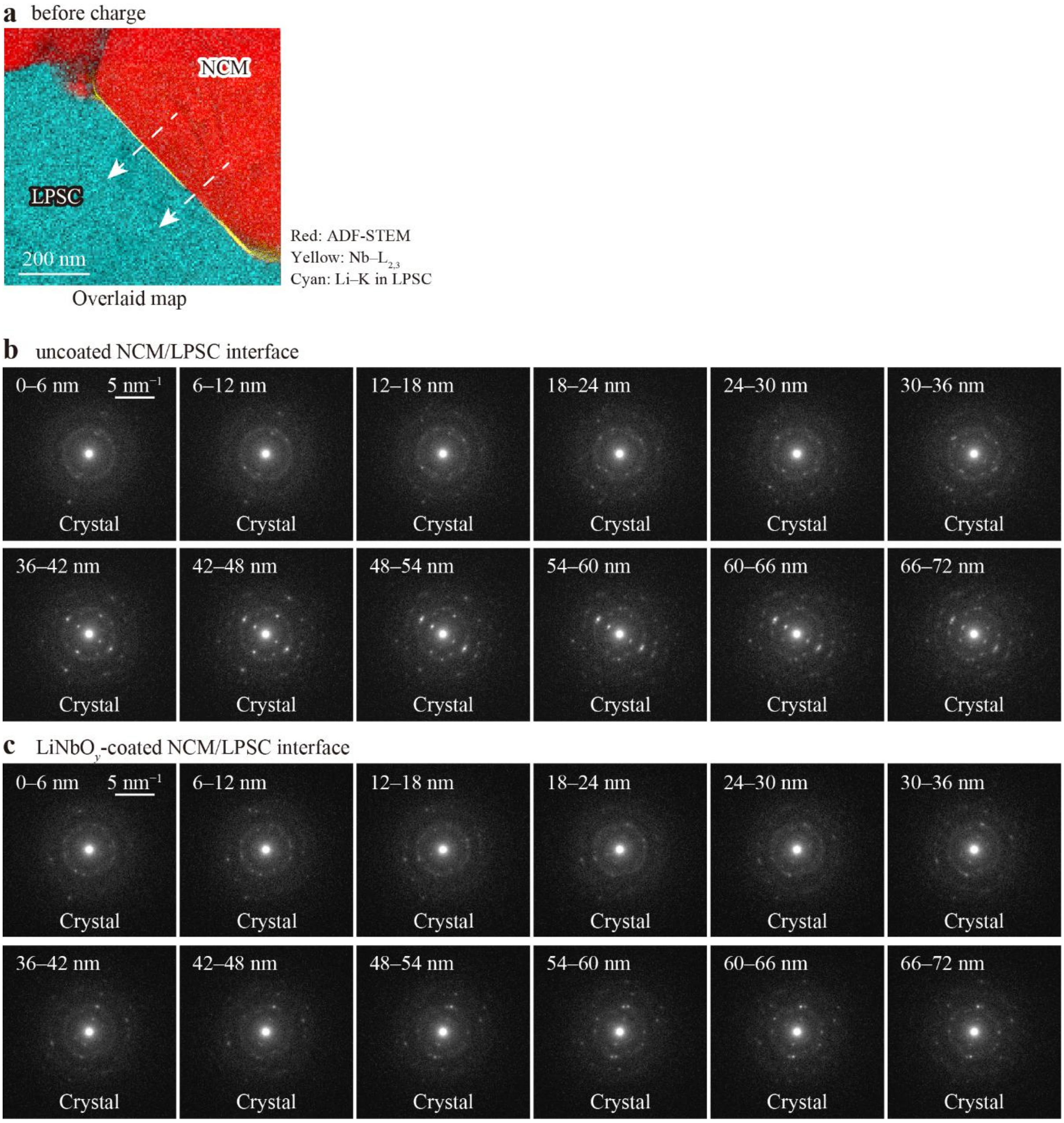

Figure S10. Crystallinity analysis ~~of~~ at the ~~CEI between~~ LiNbO$_y$-coated -NCM523 ~~and~~ /LPSC interface before charging. (a) Overlaid maps of ADF-STEM (red), Nb ~~L$_{2,3}$~~ distribution (yellow), and Li distributions in LPSC (cyan) before charge. (b) NBD patterns collected near the interface between the uncoated NCM523 and LPSC. (c) NBD patterns collected near the interface between the LiNbO$_y$-coated (5 nm thick) NCM523 and LPSC. Note that these NBD patterns were acquired without the energy filter, resulting in a stronger diffused background due to inelastic scattering as compared to those shown in Figure 4 and 6.

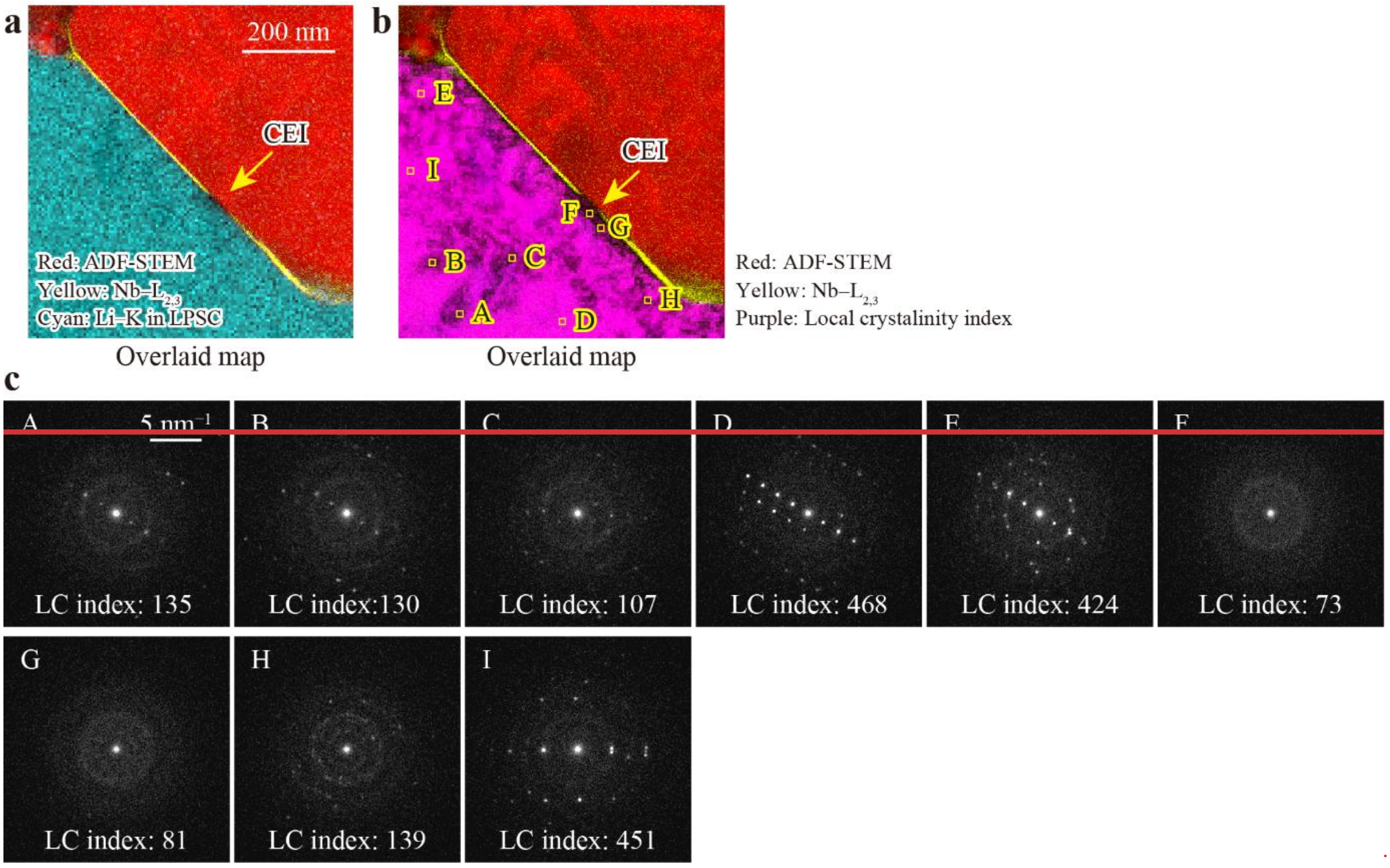

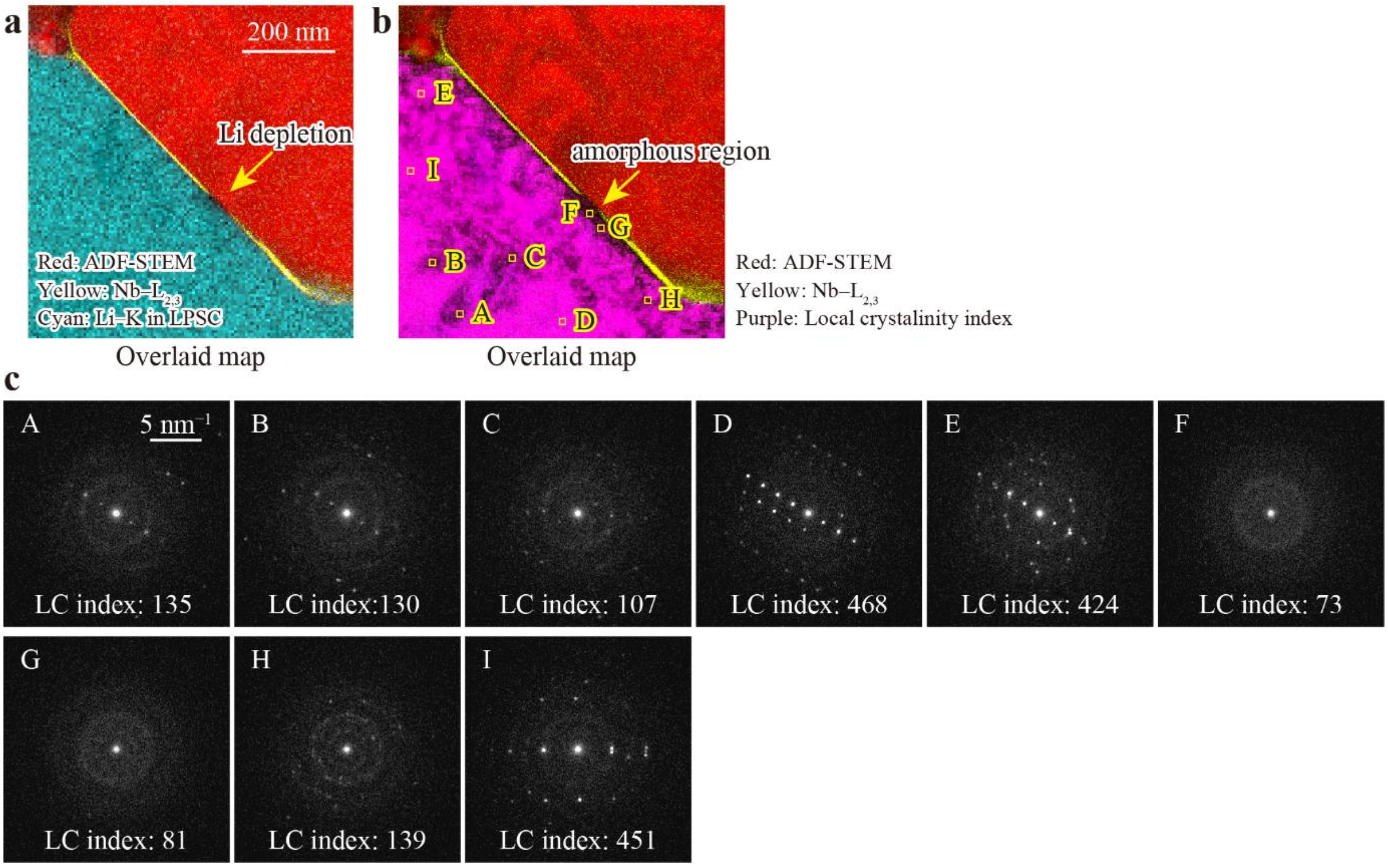

Figure S11. *LC* analysis at the CEI between LiNbO$_y$-coated NCM523 and /LPSC interface. (a) Overlaid map of ADF-STEM (red), Nb–$L_{2,3}$ distribution, (yellow), and Li distribution in LPSC (cyan). (b) Overlaid map of ADF-STEM (red), Nb–$L_{2,3}$ distribution (yellow), and *LC* index (purple), where higher *LC* values indicate higher crystallinity. (c) EF-NBD patterns collected at the positions A–I that are shown in (b).

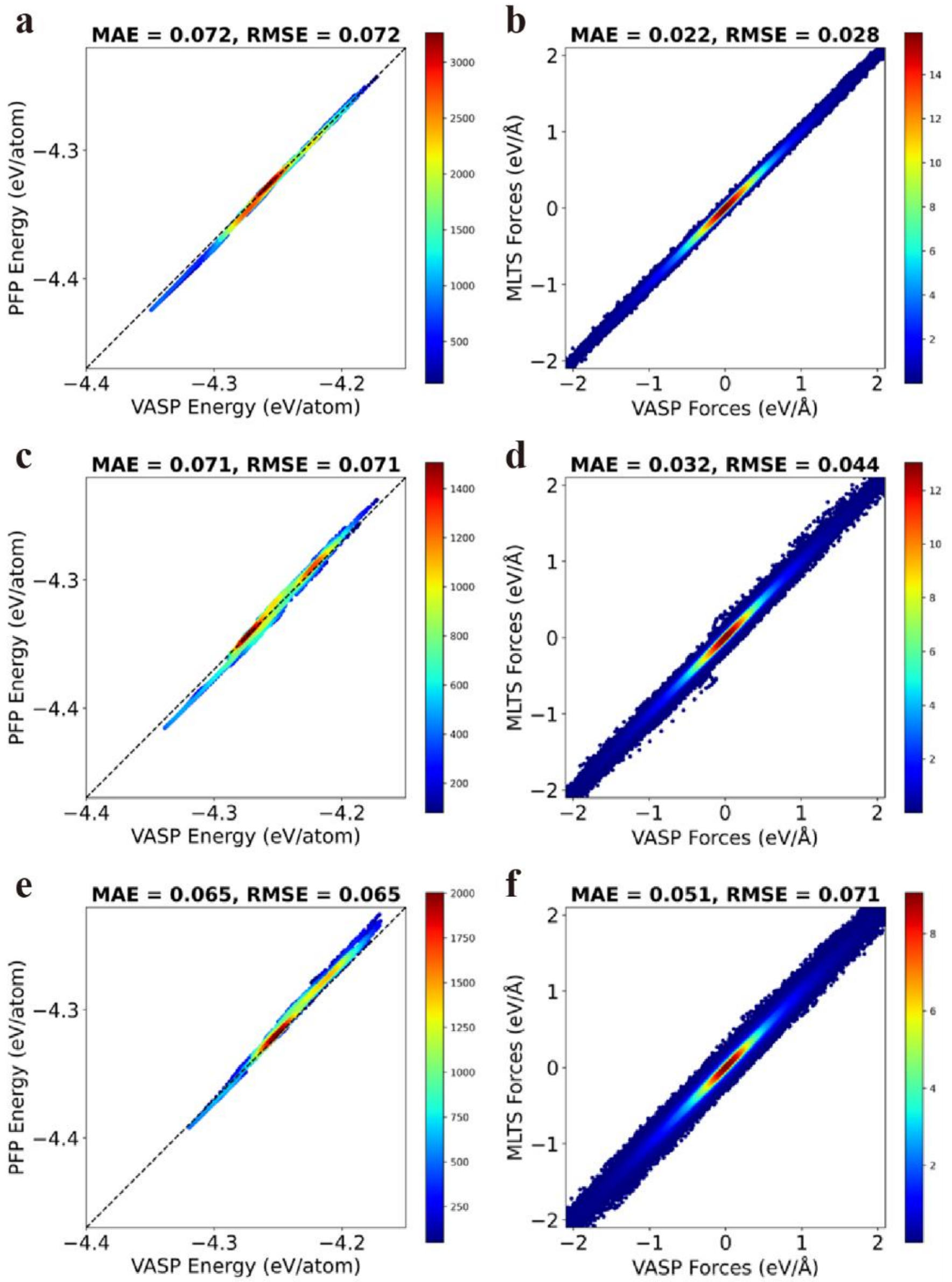

Figure S12. Validation of the NNP-PFP potential against DFT calculations for Li-deficient Li_xPS_5Cl structures. (a, b) Li_6PS_5Cl; (c, d) Li_4PS_5Cl; (e, f) Li_2PS_5Cl. Panels (a), (c), and (e) compare the energies per atom, whereas panels (b), (d), and (f) compare the atomic forces.

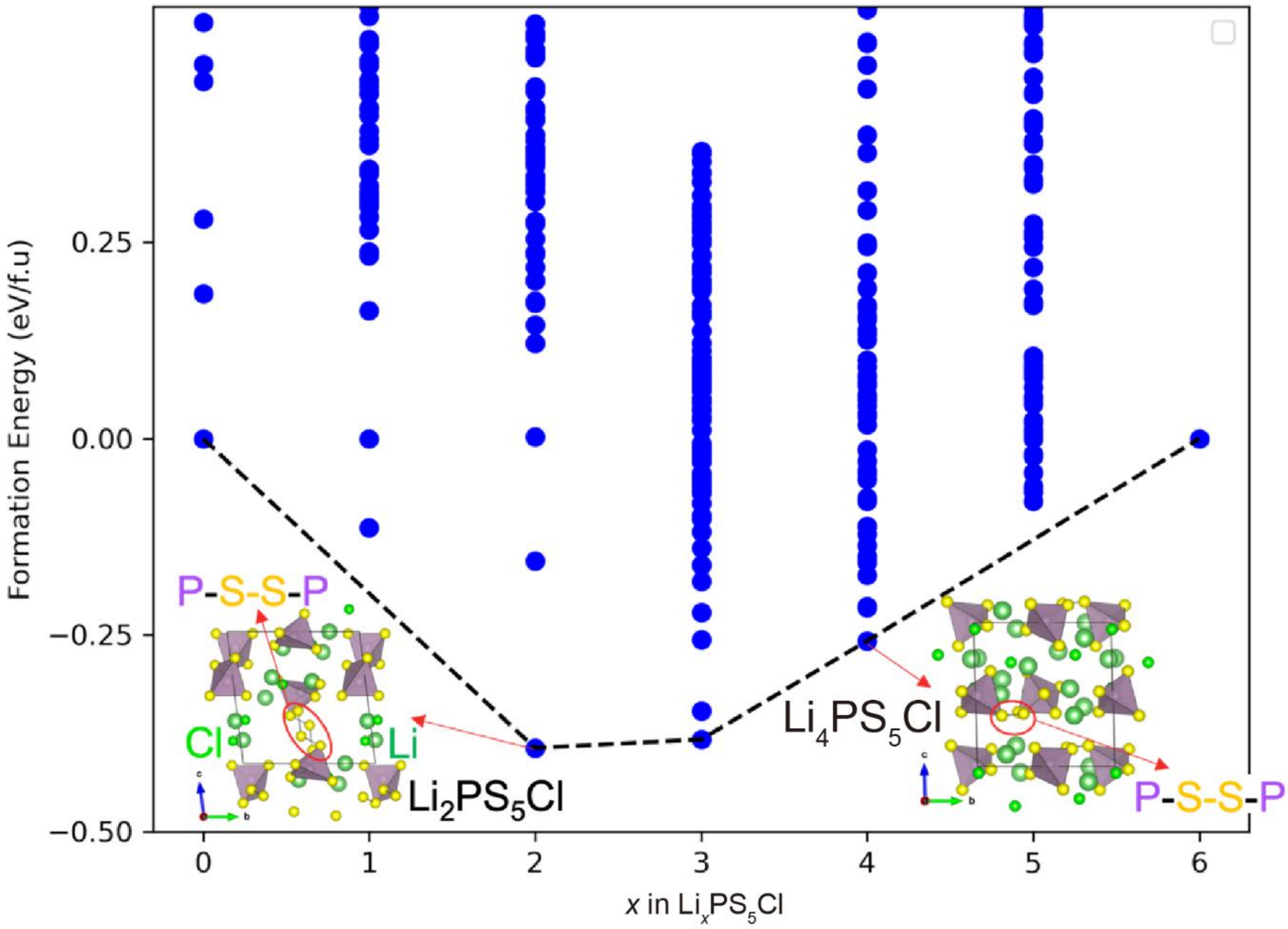

Figure S13. Convex hull obtained by DFT simulation. Li_6PS_5Cl and PS_5Cl are chosen as the references using the following equation: $\Delta H = E_{\mathrm{Li_xPS_5Cl}} - \frac{x}{6}E_{\mathrm{Li_6PS_5Cl}} - (1-\frac{x}{6})E_{\mathrm{PS_5Cl}}$, where ΔH is formation energy and E_A is the total energy of structure A. The result indicates the lowest energy structures for Li_2PS_5Cl (left) and Li_4PS_5Cl (right) after DFT optimization.

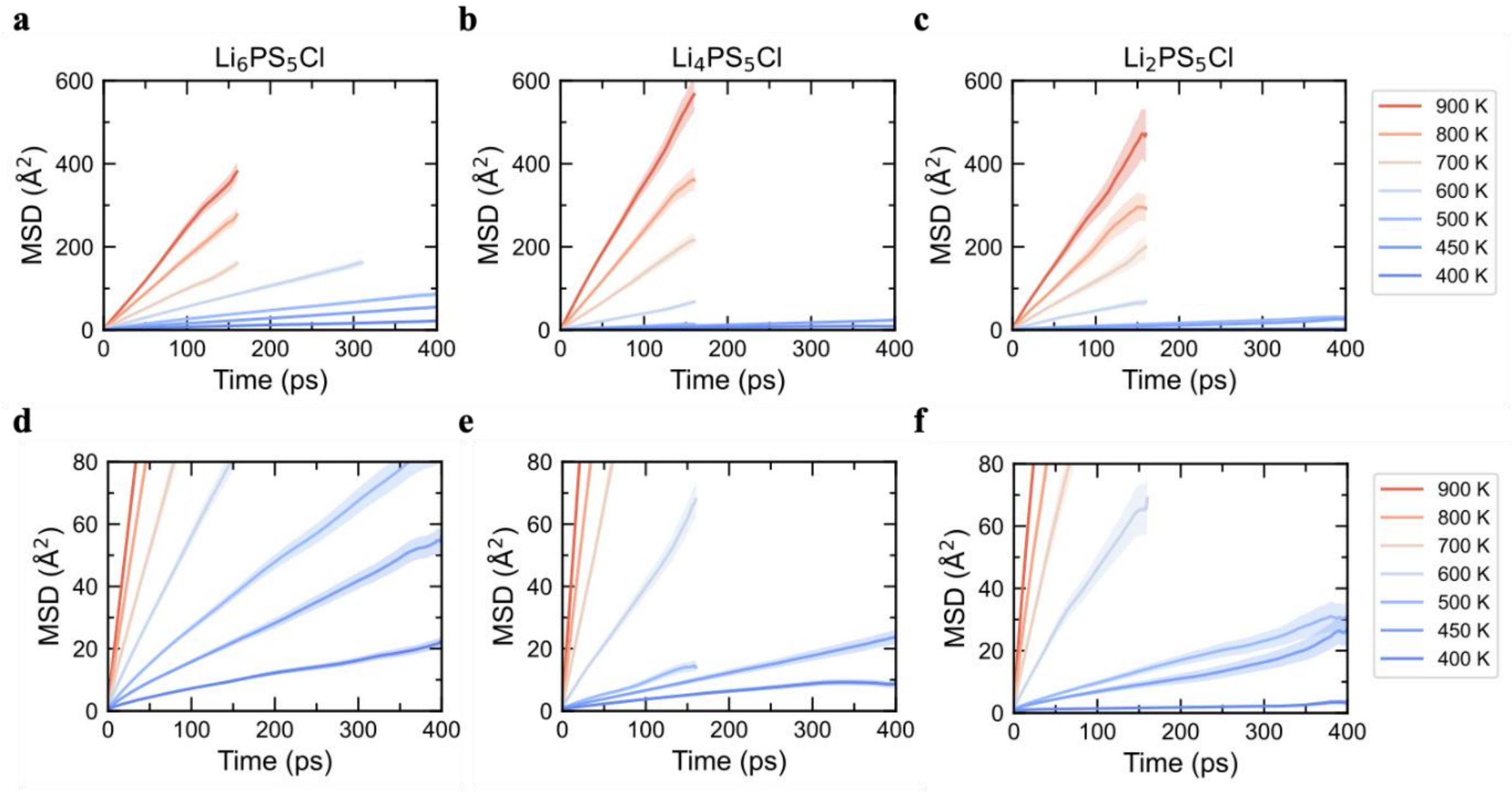

Figure S14. Mean-squared displacement (MSD) of Li ions in Li_xPS_5Cl at various temperatures obtained from MD simulations. (a–c) Overall MSD as a function of the simulation time for Li_6PS_5Cl, Li_4PS_5Cl, and Li_2PS_5Cl, respectively. (d–f) Corresponding MSD plots with a reduced vertical axis range to highlight the MSD regime at lower temperatures.

References

[1] W. S. K. Bong, A. Shiota, T. Miwa, Y. Morino, S. Kanada, K. Kawamoto, *J. Power Sources* **2023**, *577*, 233259.
[2] N. Minafra, M. A. Kraft, T. Bernges, C. Li, R. Schlem, B. J. Morgan, W. G. Zeier, *Inorg. Chem.* **2020**, *59*, 11009.
[3] S. Takamoto, C. Shinagawa, D. Motoki, K. Nakago, W. Li, I. Kurata, T. Watanabe, Y. Yayama, H. Iriguchi, Y. Asano, T. Onodera, T. Ishii, T. Kudo, H. Ono, R. Sawada, R. Ishitani, M. Ong, T. Yamaguchi, T. Kataoka, A. Hayashi, N. Charoenphakdee, T. Ibuka, *Nat. Commun.* **2022**, *13*, 2991.
[4] S. Grimme, J. Antony, S. Ehrlich, H. Krieg, *J. Chem. Phys.* **2010**, *132*, 154104.
[5] J. P. Perdew, K. Burke, M. Ernzerhof, *Phys. Rev. Lett.* **1996**, *77*, 3865.
[6] G. Kresse, J. Hafner, *Phys. Rev. B* **1993**, *47*, 558.
[7] G. Kresse, D. Joubert, *Phys. Rev. B* **1999**, *59*, 1758.
[8] M. Parrinello, A. Rahman, *Phys. Rev. Lett.* **1980**, *45*, 1196.
[9] S. Nosé, *Mol. Phys.* **1984**, *52*, 255.
[10] W. G. Hoover, *Phys. Rev. A* **1985**, *31*, 1695.
[11] A. R. McCluskey, A. G. Squires, J. Dunn, S. W. Coles, B. J. Morgan, *J. Open Source Softw.* **2024**, *9*, 5984.
[12] A. R. McCluskey, S. W. Coles, B. J. Morgan, *J. Chem. Theory Comput.* **2025**, *21*, 79.
[13] F. Walther, R. Koerver, T. Fuchs, S. Ohno, J. Sann, M. Rohnke, W. G. Zeier, J. Janek, *Chem. Mater.* **2019**, *31*, 3745.
[14] Y. Yamagishi, H. Morita, Y. Nomura, E. Igaki, *ACS Appl. Mater. Interfaces* **2021**, *13*, 580.
[15] Y. Tang, X.-Y. Wang, J.-C. Ren, B.-W. Chen, Z.-Y. Huang, W. Wang, Y.-L. Huang, B.-H. Zhang, S. Lan, Z.-L. He, Q. Liu, H. He, *Rare Metals* **2024**, *43*, 41.
[16] K. Yoshikawa, T. Kato, Y. Suzuki, A. Shiota, T. Ohnishi, K. Amezawa, A. Nakao, T. Yajima, Y. Iriyama, *Adv. Sci.* **2024**, *11*, e2402528.
[17] R. Koerver, I. Aygün, T. Leichtweiß, C. Dietrich, W. Zhang, J. O. Binder, P. Hartmann, W. G. Zeier, J. Janek, *Chem. Mater.* **2017**, *29*, 5574.~~[1] W. S. K. Bong, A. Shiota, T. Miwa, Y. Morino, S. Kanada, K. Kawamoto, *J. Power Sources* **2023**, *577*, 233259.~~
~~[2] N. Minafra, M. A. Kraft, T. Bernges, C. Li, R. Schlem, B. J. Morgan, W. G. Zeier, *Inorg. Chem.* **2020**, *59*, 11009.~~
~~[3] S. Takamoto, C. Shinagawa, D. Motoki, K. Nakago, W. Li, I. Kurata, T. Watanabe, Y. Yayama, H. Iriguchi, Y. Asano, T. Onodera, T. Ishii, T. Kudo, H. Ono, R. Sawada, R. Ishitani, M. Ong, T. Yamaguchi, T. Kataoka, A. Hayashi, N. Charoenphakdee, T. Ibuka, *Nat. Commun.* **2022**, *13*, 2991.~~
~~[4] S. Grimme, J. Antony, S. Ehrlich, H. Krieg, *J. Chem. Phys.* **2010**, *132*, 154104.~~
~~[5] J. P. Perdew, K. Burke, M. Ernzerhof, *Phys. Rev. Lett.* **1996**, *77*, 3865.~~
~~[6] G. Kresse, J. Hafner, *Phys. Rev. B* **1993**, *47*, 558.~~
~~[7] G. Kresse, D. Joubert, *Phys. Rev. B* **1999**, *59*, 1758.~~
~~[8] M. Parrinello, A. Rahman, *Phys. Rev. Lett.* **1980**, *45*, 1196.~~
~~[9] S. Nosé, *Mol. Phys.* **1984**, *52*, 255.~~
~~[10] W. G. Hoover, *Phys. Rev. A* **1985**, *31*, 1695.~~

[11] A. R. McCluskey, A. G. Squires, J. Dunn, S. W. Coles, B. J. Morgan, *J. Open Source Softw.* **2024**, *9*, 5984.

[12] A. R. McCluskey, S. W. Coles, B. J. Morgan, *J. Chem. Theory Comput.* **2025**, *21*, 79.

[13] F. Walther, R. Koerver, T. Fuchs, S. Ohno, J. Sann, M. Rohnke, W. G. Zeier, J. Janek, *Chem. Mater.* **2019**, *31*, 3745.

[14] Y. Yamagishi, H. Morita, Y. Nomura, E. Igaki, *ACS Appl. Mater. Interfaces* **2021**, *13*, 580.

[15] Y. Tang, X.-Y. Wang, J.-C. Ren, B.-W. Chen, Z.-Y. Huang, W. Wang, Y.-L. Huang, B.-H. Zhang, S. Lan, Z.-L. He, Q. Liu, H. He, *Rare Metals* **2024**, *43*, 41.

[16] K. Yoshikawa, T. Kato, Y. Suzuki, A. Shiota, T. Ohnishi, K. Amezawa, A. Nakao, T. Yajima, Y. Iriyama, *Adv. Sci.* **2024**, *11*, e2402528.

[17] R. Koerver, I. Aygün, T. Leichtweiß, C. Dietrich, W. Zhang, J. O. Binder, P. Hartmann, W. G. Zeier, J. Janek, *Chem. Mater.* **2017**, *29*, 5574.
